# Distinct B cell subsets in Peyer’s patches convey probiotic effects by *Limosilactobacillus reuteri*

**DOI:** 10.1186/s40168-021-01128-4

**Published:** 2021-10-03

**Authors:** Hao-Yu Liu, Antoine Giraud, Cedric Seignez, David Ahl, Feilong Guo, John Sedin, Tomas Walden, Jee-Hwan Oh, Jan Peter van Pijkeren, Lena Holm, Stefan Roos, Stefan Bertilsson, Mia Phillipson

**Affiliations:** 1 Laboratory of Animal Physiology and Molecular Nutrition, College of Animal Science and Technology, Yangzhou University, 225009 Yangzhou, PR China; 2 Department of Medical Cell Biology, Uppsala University, PO box 571, 75123 Uppsala, Sweden; 3 Department of Food Science, University of Wisconsin-Madison, Madison, WI 53706 USA; 4 Department of Molecular Sciences, Swedish University of Agricultural Sciences, 75007 Uppsala, Sweden; 5 Department of Aquatic Sciences and Assessment, Swedish University of Agricultural Sciences, 75651 Uppsala, Sweden; 6 Science for Life Laboratory, 75237 Uppsala, Sweden

**Keywords:** Innate-like B lymphocytes, Inflammatory bowel disease, Gut microbiome, PD-1 dependent, Probiotics, R2LC

## Abstract

**Background:**

Intestinal Peyer’s patches (PPs) form unique niches for bacteria-immune cell interactions that direct host immunity and shape the microbiome. Here we investigate how peroral administration of probiotic bacterium *Limosilactobacillus reuteri* R2LC affects B lymphocytes and IgA induction in the PPs, as well as the downstream consequences on intestinal microbiota and susceptibility to inflammation.

**Results:**

The B cells of PPs were separated by size to circumvent activation-dependent cell identification biases due to dynamic expression of markers, which resulted in two phenotypically, transcriptionally, and spatially distinct subsets: small IgD^+^/GL7^−^/S1PR1^+^/*Bcl6*, *CCR6*-expressing pre-germinal center (GC)-like B cells with innate-like functions located subepithelially, and large GL7^+^/S1PR1^−^/Ki67^+^/*Bcl6*, *CD69-*expressing B cells with strong metabolic activity found in the GC. Peroral *L. reuteri* administration expanded both B cell subsets and enhanced the innate-like properties of pre-GC-like B cells while retaining them in the sub-epithelial compartment by increased sphingosine-1-phosphate/S1PR1 signaling. Furthermore, *L. reuteri* promoted GC-like B cell differentiation, which involved expansion of the GC area and autocrine TGFβ-1 activation. Consequently, PD-1-T follicular helper cell-dependent IgA induction and production was increased by *L. reuteri*, which shifted the intestinal microbiome and protected against dextran-sulfate-sodium induced colitis and dysbiosis.

**Conclusions:**

The Peyer’s patches sense, enhance and transmit probiotic signals by increasing the numbers and effector functions of distinct B cell subsets, resulting in increased IgA production, altered intestinal microbiota, and protection against inflammation.

Video abstract

**Supplementary Information:**

The online version contains supplementary material available at 10.1186/s40168-021-01128-4.

## Background

Interactions between the gut microbiota and the immune system are important for host physiology and susceptibility to disease, but also for the therapeutic responses to cancer immunotherapies [1–3]. Accumulating evidence reveals that specific bacterial taxa can regulate the differentiation and function of intestinal immune cells [2, 4]. *Limosilactobacillus reuteri* (previously named *Lactobacillus reuteri*) [5] is such a host-specific symbiont that is highly diversified and adapted to the mammalian and avian gastrointestinal (GI) tract [6]. Multiple beneficial effects of *L. reuteri* have been demonstrated both in clinical and preclinical studies, including prevention and/or amelioration of inflammatory diseases and GI tract disorders [7]. Using the dextran sulfate sodium (DSS)-colitis model, we have previously demonstrated that *L. reuteri* R2LC treatment strengthens the intestinal mucosal barrier and represses inflammation [8]. These observations justify attempts to use probiotic bacteria for manipulating the host immune system to maintain intestinal homeostasis and combat inflammation, as well as to enhance the efficacies of tumor-targeting treatments [3]. In addition to influencing the host, the intestinal microbiota is shaped by the host through a multi-layer system that involves epithelium, antibacterial molecules, innate and adaptive immune cells, as well as immunoglobulin A (IgA) antibodies secreted by plasma cells interspersed throughout the mucosa [9, 10]. Absence or impaired selection of IgA causes gut microbiota dysbiosis and leads to systemic immune effects and overt inflammation [11].

Induction of IgA primarily takes place in the germinal centers (GCs) of the intestinal lymphoid tissue, the Peyer’s patches (PPs) [12]. The PPs are in direct contact with luminal antigens that are captured at the follicle-associated-epithelium (FAE) for subsequent transport to the sub-epithelial dome (SED) occupied by antigen-presenting dendritic cells (DCs) and numerous B lymphocytes [12–14]. Activated IgD^+^ pre-GC B cells in the SED are reported to express CCR6 and interact with DCs to obtain signals for IgA induction [12, 15]. The DCs serve as an important source of transforming growth factor β (TGFβ), the key factor that drives IgA class switch recombination [16, 17] and supports the B cell IgA-switch in the SED [13]. However, the dominant part of IgA induction occurs in the GC regions of the PPs [10], where the GC reactions constantly occur due to the continuous exposure of PPs to gut microbiota [12, 14]. Here, proliferating Ki67^+^, GL7^+^ GC B cells express activation-induced cytidine deaminase (AICDA) and BCL6, which are critical factors for antibody clonal expansion and affinity maturation [10, 12]. Despite recent progress within the field, a detailed understanding of the interactions between intestinal microbiota and the immune cells of PPs is still lacking.

Here we explore how the probiotic bacterium *L. reuteri* R2LC affects the B cells of PPs and consecutive intestinal IgA induction and production, as well as the downstream consequences on intestinal microbiota and susceptibility to inflammation. To limit possible biases of cell identification by their dynamic expression of surface markers, the CD19^+^B220^+^ PPs B lymphocytes were sorted by size. This resulted in two phenotypically distinct subsets that were transcriptionally and spatially separated: large GC-like B cells of the GC area, and smaller pre-GC B cells with an innate-like phenotype residing sub-epithelially. Following peroral administration of *L. reuteri* R2LC for seven days, both population sizes expanded and their respective intrinsic functions were strengthened. Ultimately, *L. reuteri* R2LC treatment modified the intestinal microbiota through enhanced IgA induction and production, as well as reduced inflammation and microbiota dysbiosis in a model of inflammatory bowel diseases (IBD).

## Results

### Phenotypic and transcriptional distinction between B cells in Peyer’s patches

**Fig. 1:**
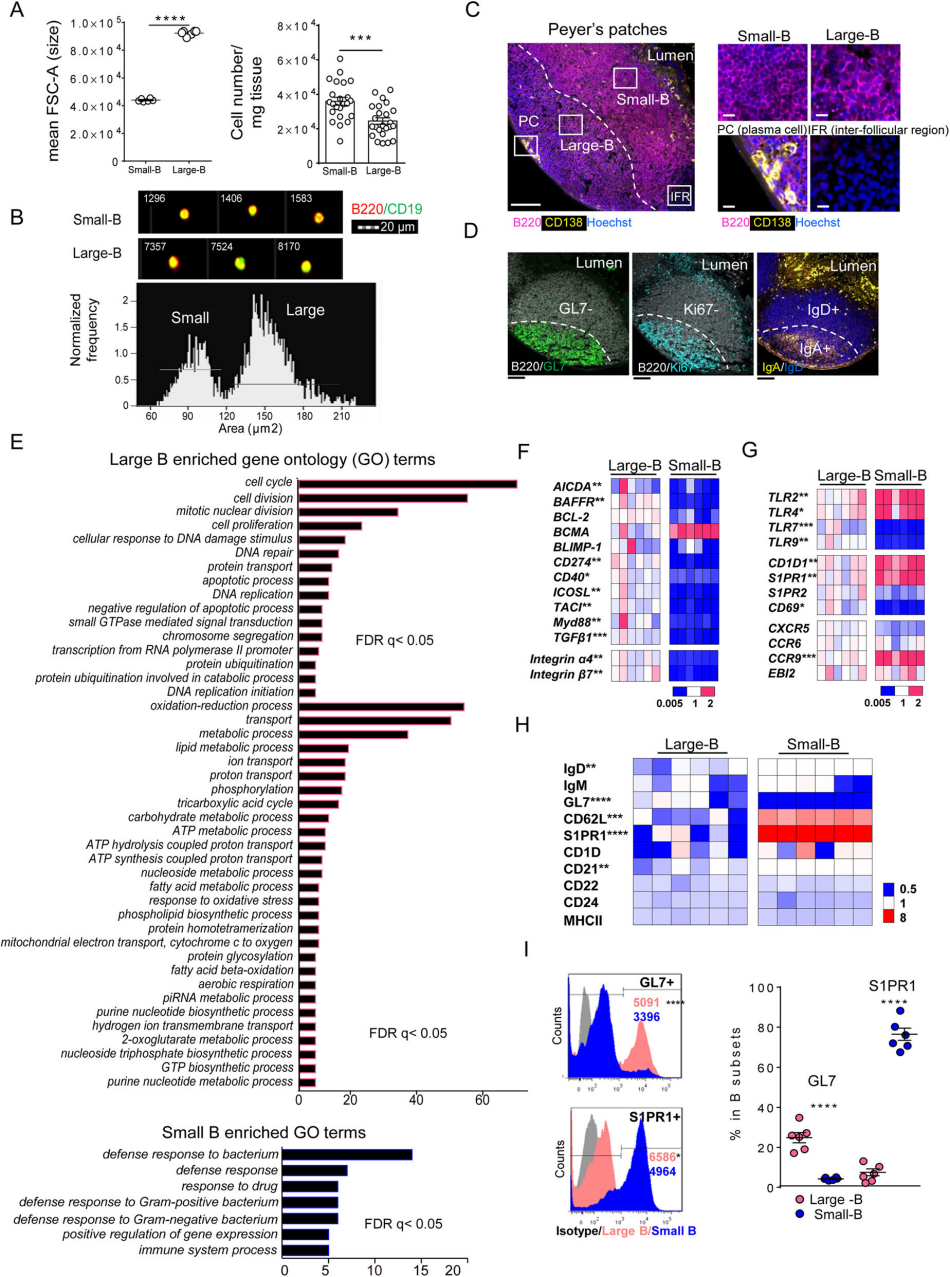
Phenotypic and transcriptional distinction between B cell subsets in Peyer’s patches. **a** Flow cytometry of PPs live CD3^-^CD19^+^B220^+^ B lymphocytes (gated as shown in Fig. S1A) and separated into the small and large B cell subsets by forward scatter area and expressed as mean FSC-A (size), and the number of small-B and large-B cells per mg tissue (*n* = 6 mice left panel; *n* = 24 mice right panel). **b** Imaging flow cytometry of the areas (μm^2^) of small-B and large-B cell from three independent experiments. **c**, **d** Immunohistochemistry of PPs stained with anti-B220 (magenta), anti-CD138 (yellow) and Hoechst (blue) in **c** or anti-B220 (white), anti-GL7 (green), anti-Ki67 (cyan), anti-IgA (yellow), and anti-IgD (blue) in **d**. Scale Bars equal 100 μm or 10 μm in closed-up reviews. **e** Enrichment of gene ontology categories (Biological Process, BP) for genes differentially expressed in large B (upper panel) versus small B cells (lower panel) determined by microarray of lin^-^CD19^+^B220^+^ cells sorted on FSC-A (*n* = 4 samples per group), the number of genes in each functional category is shown. Data were adjusted by false discovery rate control (FDR). **f, g** Displays of heat maps of genes expression by q-RT-PCR in sorted small and large B cells. The expression was normalized to the mean value of large B and each column represents one sample. **h**, **i** Heat maps and histograms depicting expression of surface markers on small and large B cells (*n* = 6 mice per group). The frequency of indicated markers (normalized to the mean value of large B cells). **i** MFI of B cells positive for GL7 and S1PR1, and percentage in each subset. Data are presented as mean ± SEM. *p < 0.05, **p < 0.01, ***p < 0.001, ****p < 0.0001 using two-tailed Student’s *t* test

The B cells of the PPs in wild-type mice were characterized on cell size using unbiased regular and imaging flow cytometry to account for potential dynamics in the expression of surface markers. We found that the CD3^-^CD19^+^B220^+^ B cells clustered into two subsets according to their size (Fig. 1a, b, Additional file 1: Fig. S1A, B), where 38.7% were large B cells (120–210 μm^2^) of higher granularity than small B cells (61.3%, 60–120 μm^2^), while size-separated clusters were not observed for the B cells from spleen or mesenteric lymph nodes (MLNs) in healthy mice. Within the PPs, immunohistochemical staining demonstrated that the two B cell subsets occupied distinct niches (Fig. 1c), where the large B cells were observed at the GL7^+^/Ki67^+^/IgA^+^ region, corresponding to the GC area [10, 15], while the small-sized B cells were predominantly found in the sub-epithelial compartment (Fig. 1c, d). Intravital microcopy confirmed that small B lymphocytes were abundant in the SED pre-GC niche [12, 14], where they were also detected interspersed between the FAE (Additional file 1: Fig. S1C, D, Additional file 2: movie S1, Additional file 3: movie S2), directly facing the luminal antigens.

Transcriptional profiling of the size-sorted PPs CD19^+^B220^+^ B cells (Additional file 1: Fig. S2A-G) revealed that both subsets expressed B cell lineage-specific transcripts (Additional file 1: Fig. S2B) and *Ig* genes in similar patterns but at different levels (Additional file 1: Fig. S2C), indicating a difference in affinity maturation [18]. Clustering analysis showed distinct subgroups of the large and small B cells (Additional file 1: Fig. S2D), where approximately 2290 genes were significantly differentially expressed (FDR *q* < 0.05). Gene ontology enrichment analysis (Biological Process) demonstrated that large B cells contain significantly more transcripts encoding proteins associated with cell cycle and proliferation, or engaged in metabolic processes including oxidation-reduction, transport, lipid oxidation, and tricarboxylic acid cycle (Fig. 1e, upper panel, Additional file 1: Table S1), especially linked with the glycolysis- and hypoxia-pathways (Additional file 1: Fig. S2F). These transcriptional programs support a high degree of biosynthesis and growth of PPs large B cells, similar to observations of the newly formed GC B cells of spleen and lymph nodes expressing the metabolic checkpoint regulators Gsk-3α (glycogen synthase kinase 3) and Hif-1α [19, 20]. Indeed, the large B cells expressed higher levels of *Mki67*, *Gsk-3α*, and *Hif-1α* compared to the small B cells (Additional file 1: Fig. S2G). Hypoxic signals were identified within the GL7^+^CD35^+^ areas using the hypoxia probe pimonidazole (Additional file 1: Fig. S2H). In addition, heat maps of mRNA expression analyzed by qRT-PCR revealed that large B cells expressed significantly higher levels of genes critical for B cell cognition and T cell interaction (*BAFFR*, *CD40*, *CD274*, *ICOSL*, and *TACI*) [21], and differentiation (*AICDA* and *TGFβ1*), *Myd88*, and *Integrin α4* and *β7* compared to small B cells (Fig. 1f).

In contrast, small B cells displayed a gene signature of innate anti-bacterial immune responses (Fig. 1e–g, Additional file 1: Table S1), involving transcripts encoding proteins such as defensins (*Defa17*, *Defa3*), lysozymes (*Lyz1*, *Lyz2*), and intestinal lactoferrin receptor (*Itln1*) (Fig. 1e, lower panel). The anti-bacterial potential of small B cells was confirmed by immunohistochemistry, as CD11C^-^CD19^+^B220^+^B cells expressing defensin α3 (DEFa3) were identified in close proximity to the high DEFa3^+^ FAE region (Additional file 1: Fig. S2I). Notably, genes of pattern recognition receptors *TLR2*, *TLR4*, the lipid presenting receptors *CD1D*, *S1PR1*, and the chemokine receptor *CCR9* were expressed at higher levels in the small compared to the large B cell subset (Fig. 1g). In contrast, the expression of nucleic acid sensing *TLR7* and *TLR9* as well as *CD69,* the negative regulator of S1PR1, were expressed at lower levels in small B cells (Fig. 1g). Both large and small B cells expressed similar levels of *Bcl6* (Additional file 1: Fig. S2G), the master regulator of GC B cell-fate [22], and genes associated with B cell migration (Fig. 1g), including *CXCR5*, *CCR6*, and *EBI2* [14, 15, 22]. Further, flow cytometry analysis showed that large B cells more frequently expressed GL7 and at higher levels (Fig. 1h, i), in contrast to the small B cells that were more often IgD^+^, CD62L^+^, CD21^+^ (*CR2*), and exclusively expressed surface S1PR1 (Fig. 1h, i). Activated B cells at pre-GC stage have been reported to express *Bcl6* [23], thus implying a potential pre-GC phenotype of the IgD^+^/GL7^−^/S1PR1^+^/*Bcl6*, *CCR6*-expressing small-B subset.

Taken together, the size of B lymphocytes in PPs is a good estimator of the B cell activation state and might therefore allow for separation and studies of specific populations in response to environmental changes. We find that large B cells in the PPs are of GC-like phenotype, expressing GL7^+^/S1PR1^−^/Ki67^+^/*Bcl6* and *CD69*, and display intense metabolic activity. In addition, small B cells represent a heterogeneous group of SED B cells that exhibit bacterial defense properties, mainly comprising of the pre-GC phenotype [22, 23], but also of IgD^+^/CD62L^+^ naïve B cells [15].

### *Limosilactobacillus reuteri* expands the different population of Peyer’s patches B cells and enhances their effector functions

**Fig. 2:**
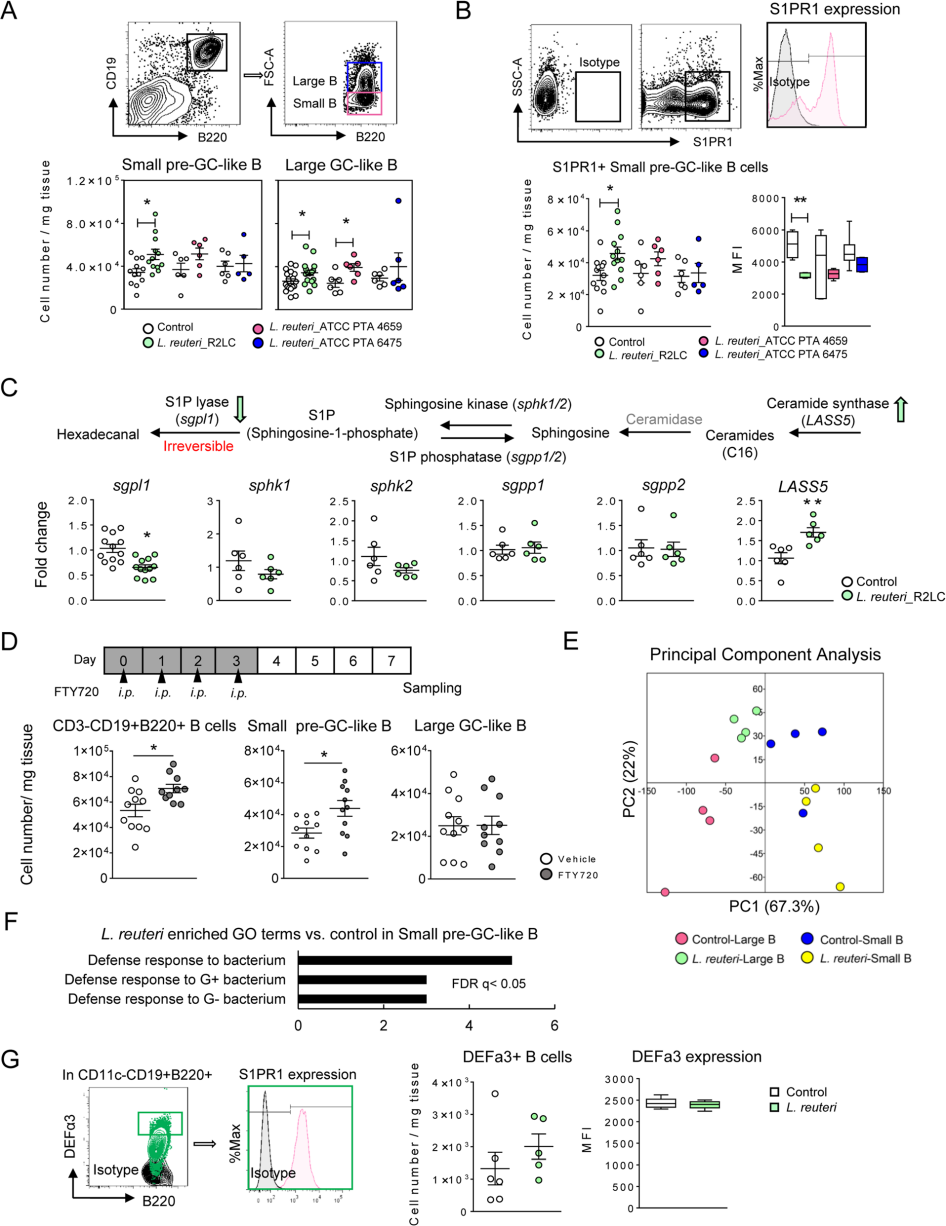
*L. reuteri* increases the population of Peyer’s patches B cells and enhances their effector functions. **a** Flow cytometry of small-pre-GC-like and large-GC-like B cell subsets from control mice or mice treated with 10^8^ cfu *L. reuteri* (R2LC, 4659 or 6475) perorally for 7 consecutive days (*n* = 5–12 mice per group). **b** Numbers of S1PR1^+^ small pre-GC-like B cells (CD3^-^CD19^+^B220^+^) and their S1PR1 expression (MFI, *n* = 5–12 mice per group). **c** Illustration of enzymes regulating sphingosine-1-phosphate (S1P) homeostasis (top). q-RT-PCR analysis of gene expression (fold change, *n* = 6 mice per group) of the S1P pathway (bottom). **d** Experimental design evaluating the effect of the sphingosine-1-phosphate (S1P) receptor modulator FTY720 (top), and flow cytometry of B cell subsets (lower panels, *n* = 10–11 mice per group). **e** Principal component analysis of the microarray data, where each dot represents one sample (*n* = 4 samples per group). **f** Enrichment of gene ontology categories (BP) for genes upregulated by *L. reuteri* in small B cells. **g** Flow cytometry of DEFa3^+^ B cells (CD11C^-^CD19^+^B220^+^) in PPs and DEFa3 expression (MFI, *n* = 5–6 mice per group). Data are presented as mean ± SEM. *p < 0.05, **p < 0.01 using two-tailed Student’s *t* test

Peroral administration of 10^8^ *L. reuteri* R2LC for seven consecutive days specifically expanded the pre-GC-like and the GC-like B cell subsets, together increasing the CD3^-^CD19^+^B220^+^ B cell population from 60 to 70% of total PPs cells (Fig. 2a, Additional file 1: Fig. S3A, B). As a result, the PPs became substantially enlarged and increased in height (Additional file 1: Fig. S3C), while the B cell population (CD3^-^CD19^+^B220^+^) in MLNs was not altered (Additional file 1: Fig. S3D), demonstrating gut-specific effects. Bacterial strain specificity was tested using two *L. reuteri* strains isolated from human breast milk (ATCC PTA 4659 and ATCC PTA 6475), whereas R2LC originates from rat intestine. While 4659 expanded only the GC-like B cell population, no difference was observed between 6475 and the control group (Fig. 2a, Additional file 1: Fig. S3B). We therefore mainly focused on R2LC, hereafter referred to as “*L. reuteri*.”

The expansion of the pre-GC-like B cells in *L. reuteri*-treated mice was almost exclusively due to increased number of S1PR1^+^ B cells (Fig. 2b) with decreased surface expression of S1PR1. These observations imply increased availability of the S1P ligand, as previously reported [24]. Indeed, *L. reuteri-*treatment down-regulated gene expression of S1P lyase (*sgpl1*) while upregulating expression of ceramide synthase (*LASS5*) in the PPs (Fig. 2c), suggesting reduced S1P degradation and amplified S1P synthesis. The involvement of the S1P/S1PR1 pathway was further assessed using a S1PR1 agonist FTY720 (Fingolimod) [24]*.* In line with the observations following *L. reuteri*-treatment, FTY720-treatment significantly increased the number of pre-GC-like, but not to GC-like B cells (Fig. 2d, Additional file 1: Fig. S3E).

Further, a principal component analysis (PCA) of the transcriptome data for the size-sorted PPs CD19^+^B220^+^ B cells from control and *L. reuteri*-treated mice revealed four separated clusters (Fig. 2e). PC1 variation confirmed the transcriptional distinction between B cells of the SED and the GC, which separated independently of bacterial stimulation. *L. reuteri*-treatment was found to impact the transcriptional profile of both subsets in two different ways: one was common for both subsets as they were shifted towards the right on PC1, whereas the other was subset-specific, as displacement on PC2 occurred in opposite directions. The differential expression of genes of large and small B cells in response to *L reuteri* treatment was further analyzed and was found to be consistent with the PCA analysis (Additional file 1: Fig. S2D), indicating distinct responses of the B cell subsets to *L. reuteri*. Gene ontology enrichment analysis of pre-GC-like B cells showed that *L. reuteri-*treatment further upregulated expression of gene clusters associated with defense responses to bacteria (Fig. 2f), including *Defa3*, *Defa21*, *Lyz1, Fbrs*, *Pira1*, *Arhgef1*, *Mptx2*, *Itln1*, *Muc2*, *Agr2*, *Mmp7*, and *Angptl1*. Although no treatment difference was observed on DEFa3^+^ protein levels in B cells, the DEFa3^+^ B cells co-expressed S1PR1 (Fig. 2g, Additional file 1: Fig. S3F). These data demonstrate that *L. reuteri* promotes the bacterial defense gene program of the S1PR1^+^ pre-GC-like B cell subset in a S1P/S1PR1-dependent manner.

### *L. reuteri* increases the numbers of B cells in the PPs GC, and modifies their functional gene signature

**Fig. 3:**
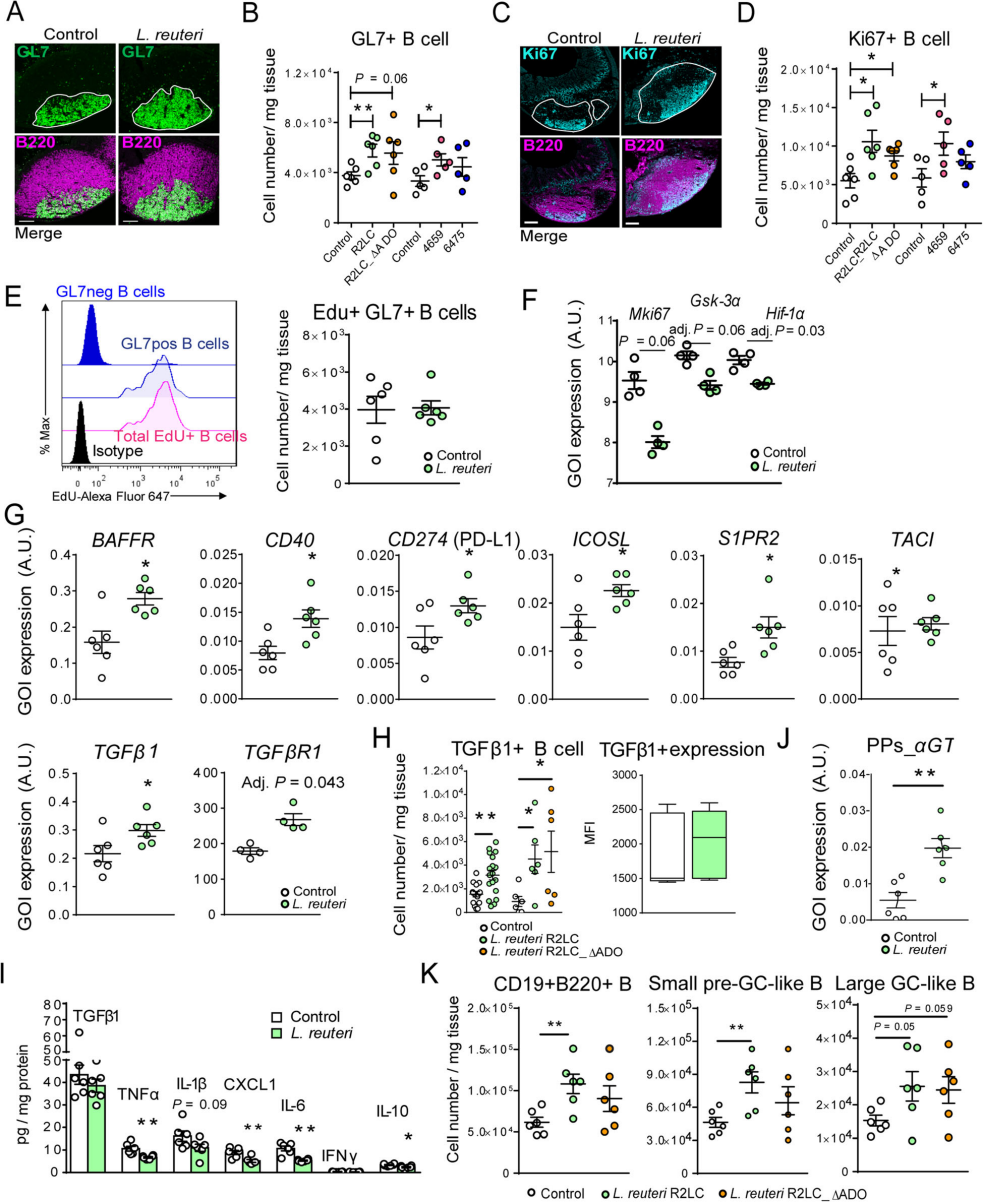
*L. reuteri* expands the GC-like B cell population and reprograms its functional gene signature. **a**, **c** Immunohistochemistry of the GL7^+^ area stained with anti-B220 (magenta), anti-GL7 (green) and anti-Ki67 (cyan). Scale bars equal 100 μm. Representative images of PPs from the control or *L. reuteri*-treatment are shown in **a** and **c**, and the corresponding quantification shown in Fig. S3G, H. **b**, **d** Flow cytometry quantification of GL7^+^ B cells (in CD3^−^CD19^+^B220^+^, *n* = 5–6 mice per group) and Ki67^+^ cells (*n* = 5–6 mice per group). **e** In vivo proliferation assay was performed by flow cytometry following *i.p*. injection of EdU. A histogram depicting EdU expression in GL7^−^, GL7^+^ or EdU^+^CD19^+^B220^+^B cells and the number of EdU^+^GL7^+^ B cells (*n* = 6 mice per group). **f** Expression of *Mki67*, *Gsk-3α*, and *Hif-1α* in large GC-like B cells from control or *L. reuteri*-treated mice (GOI, gene of interest; A.U., arbitrary unit). **g** q-RT-PCR analysis of gene expression levels (*n* = 6 mice per group) and expression of *TGFβR1* (microarray analysis). **h** Flow cytometry analyzing the numbers of TGFβ1^+^ B cells as well as the TGFβ1 levels (MFI, *n* = 6–18 mice per group). **i** The PPs tissue was analyzed for cytokine/chemokine production by Multi-Plex Mesoscale and ELISA, normalized to tissue protein content (*n* = 6 mice per group). **j** Expression of α germline transcripts (*αGT*) in PPs (*n* = 6 mice per group). **k** The numbers of B cells of different subsets in PPs in untreated mice and in response to *L. reuteri* R2LC and *L. reuteri* R2LC_ΔADO (*n* = 6 mice per group). Data are presented as mean ± SEM. *p < 0.05, **p < 0.01 using Student’s *t* test or ANOVA with Tukey’s post hoc test

*L. reuteri*-treatment expanded the GL7^+^ GC area of the PPs (Fig. 3a*,* Additional file 1: Fig. S3G), where more proliferating Ki67^+^ B cells were found to accumulate (Fig. 3c*,* Additional file 1: Fig. S3H). Flow cytometry confirmed that *L. reuteri* (R2LC and 4659) increased the number of GL7^+^ and Ki67^+^ B cells (Fig. 3b, d). In addition, active DNA synthesis determined by EdU (5-ethynyl-2′-deoxyuridine) uptake on the last day of the experiment occurred in GL7^+^ but not GL7^-^ B cells (Fig. 3e, left panel), whereas the number of EdU^+^GL7^+^B cells was unaltered (Fig. 3e, right panel). In contrast, transcriptome analysis of the GC-like B cells showed that *L. reuteri*-treatment downregulated cell division and cell cycle gene clusters (Additional file 1: Table S2), and expression of *Mki67*, *Gsk-3α*, and *Hif-1α* (Fig. 3f). Moreover, the proportion of HIF-1α^+^ B cells and their HIF-1α expression were reduced by *L. reuteri-*treatment (Additional file 1: Fig. S3I). These results indicate that *L. reuteri* stimulate the proliferation and clustering of GC-like B cells at the GC niche. Furthermore, *L. reuteri*-treatment upregulated the gene signature of GC function in these B cells (Fig. 3g), and expressions of *TGFβ1* and *TGFβR1*. Using flow cytometry, we discovered a significant expansion of TGFβ1^+^ B cells in PPs of *L. reuteri*-treated mice (Fig. 3h). The *L. reuteri*-induction of TGFβ1 was specific to the *TGFβ1-TGFβR1-*expressing GC-like B cell subset, and the tissue concentration of TGFβ1 did not change (Fig. 3i). In contrast, *L. reuteri*-treatment reduced cytokine levels in the PPs including TNF-α, CXCL1, IL-6, and IL-10 (Fig. 3i), promoting a non-inflammatory shift of the microenvironment. In addition, *L. reuteri*-treatment upregulated the IgA germline transcript (*αGT*) in PPs (Fig. 3j). These data demonstrate that *L. reuteri* enhances the functional gene expression of GC-like B cells, and promotes their low proliferative/high T cell-interactive post-GC phenotype [21] by autocrine stimulation of TGFβ1 signaling [15].

*L. reuteri* DSM 17938 has previously been reported to modulate gut microbiome and inhibit autoimmune responses in immune-compromised Scurfy mice via the adenosine receptor A_2A_ [25]. To determine whether *L. reuteri* R2LC induces PPs B cell responses through adenosine signaling, we generated a mutant strain lacking the ability to convert AMP into adenosine by impaired functional 5′-nucleotidase (*L. reuteri* R2LC_ΔADO). The effect of the mutant on the PPs B cells subsets did not differ in any respect from the wild-type R2LC strain (Fig. 3b, d, h, k). However, the mutant increased the number of Ki67^+^ and TGFβ1^+^ B cells in the PPs compared to control mice (Fig. 3d, h). These results indicate that adenosine signaling alone does not determine the effects by R2LC on PPs B cells.

### *L. reuteri* promotes B cell IgA-responses through the T follicular helper (Tfh)-PD-1 pathway

**Fig. 4:**
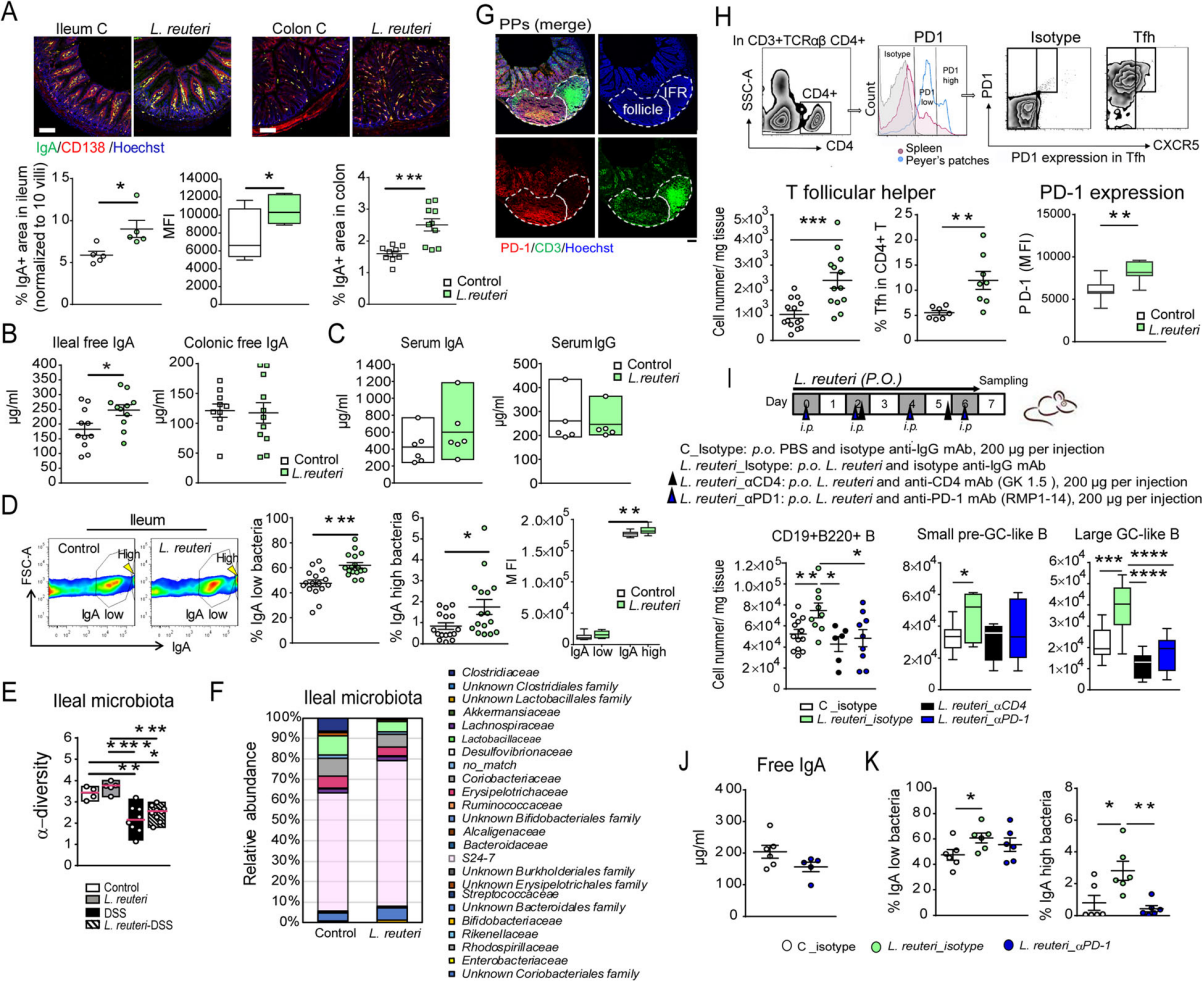
*L. reuteri* promotes B cell IgA-responses in a Tfh-PD-1-dependent manner. **a** The area of IgA^+^ plasma cells (anti-CD138) quantified in ileal and colonic tissues, *n* = 5 mice per group (duplicate slides per mouse, scale bars equal 100 μm). **b** Free IgA (μg/ml, ELISA) in the lumen of ileum and colon (*n* = 10–11 mice per group) and **c** Serum IgA and IgG concentrations (μg/ml, ELISA, *n* = 5-6 mice per group). **d** Flow cytometry of IgA^+^ bacteria in ileum (*n* = 16-17 mice per group). **e**, **f** Ileal microbiome assessed by 16S rRNA gene amplicon sequencing. Microbial community diversity was calculated as α-diversity in mice treated with *L. reuteri* and/or DSS compared to controls, *n* = 4–9 mice per group. Data are presented as median values. **g** Localization of PD-1^+^ T cells in PPs stained with anti-PD-1 (red), anti-CD3 (green), and Hoechst (blue, scale bar equals 100 μm) from three independent experiments. **h** Flow cytometry of Tfh cells and PD-1 expression (*n* = 13-14 mice per group). A histogram of PPs and spleen indicating a uniquely high expression of PD-1 in PPs T cells. **i** Design of PD-1 monoclonal antibodies blocking (blue arrows) and CD4^+^ T cell depletion (black arrows) experiments and effects on the number of B cell subsets (*n* = 6–13 mice per group). **j**, **k** Free IgA production and quantification of IgA^+^ bacteria in the ileum from PD-1 (mAb) blocking experiments (*n* = 6 mice per group). Data are presented as mean ± SEM*.* *p < 0.05, **p < 0.01, ***p < 0.001, ****p < 0.*0001* using two-tailed Student’s *t* test or ANOVA with Tukey’s post hoc test

As *L. reuteri* was demonstrated to enhance IgA induction in PPs, its effects on IgA production and secretion were investigated. Interestingly, we found that *L. reuteri*-treatment increased the density of IgA^+^ plasma cells in the lamina propria of both colon and ileum, as well as increased IgA expression in the ileum (Fig. 4a). Accordingly, higher titers of free IgA were detected in the intestinal lumen, whereas IgA or IgG concentrations in circulation were not altered (Fig. 4b, c). Following secretion, IgA serves as part of innate immune surveillance through binding commensal microbiota [26]. Flow cytometry revealed that *L. reuteri*-treatment enhanced the proportion of IgA^+^ bacteria in the ileal flora, as well as the level of IgA bound per bacteria (IgA^high^) (Fig. 4d, Additional file 1: Fig. S4A). Increased number of IgA^high^ bacteria was also observed with *L. reuteri* R2LC_ΔADO compared to control (Additional file 1: Fig. S4B). *L. reuteri*-treatment did not increase the α-diversity of the microbiota (Fig. 4e, Additional file 1: Fig. S4C), but induced a shift of bacterial community composition (Fig. 4f, Additional file 1: Fig. S4D). Notably, ileal *Clostridiaceae*, *Erysipelotrichaceae* and other bacteria belonging to *Firmicutes* were less abundant in *L. reuteri-*treated mice compared to in control mice (Additional file 1: Fig. S4E). In contrast, *L. reuteri*-treatment promoted the expansion of ileal *Bifidobacteriaceae*, as well as ileal and colonic bacterial phylotype *S24*-7 (affiliated to *Bacteroidetes*) (Fig. 4f, Additional file 1: Fig. S4D, E), while the relative abundance of *Lactobacillaceae* did not increase at either site. Together these results demonstrate that administration of a single species of *L. reuteri* boosts IgA production, thereby shifts the composition of the intestinal microbiota.

In lymphoid organs, PD-1^+^CXCR5^+^ Tfh cells are specialized in promoting T cell-dependent B cell IgA-responses [21]. Immunohistochemical staining revealed that in PPs, the PD-1^+^ Tfh cells are localized in the GC area (Fig. 4g). *L. reuteri*-treatment did not affect the CD3^+^TCRαβ^+^CD4^+^ T cell population (Additional file 1: Fig. S4F); however it doubled the number of PD-1^+^CXCR5^high^ Tfh cells, which expressed higher levels of PD-1 (Fig. 4h) and increased the number of IL-21^+^CD4^+^ effector T cells and their IL-21 production (Additional file 1: Fig. S4F). Importantly, the *L reuteri*-induced expansion of GC-like B cells was completely dependent on the Tfh-PD-1 pathway, as demonstrated by either administration of PD-1-blocking monoclonal antibodies or depletion of CD4^+^ cells (Fig. 4i). PD-1 inhibition also impaired the ability of *L. reuteri*-treatment to enhance IgA production, resulting in reduced levels of IgA-binding bacteria (Fig. 4j, k). These data demonstrate that the Tfh-PD-1 pathway is crucial for *L. reuteri*-induction of B cell IgA-responses.

### *L. reuteri* provides protection against DSS-induced intestinal inflammation and disruption of Peyer’s patches

**Fig. 5:**
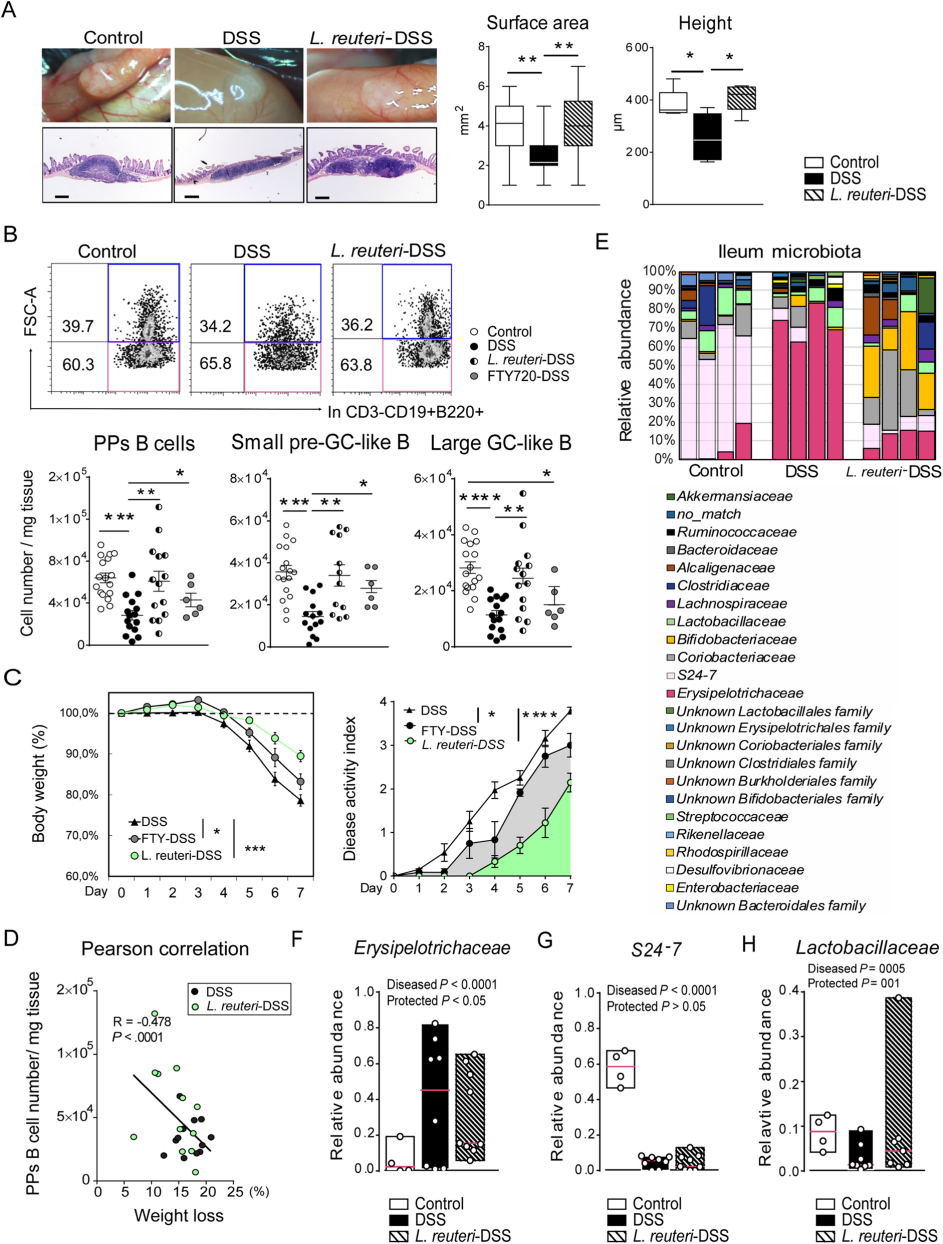
*L. reuteri* provides protection against DSS-induced colitis, disruption of Peyer’s patches and intestinal dysbiosis. **a** Macroscopic analysis of the PPs surface area (mean area of PPs per mouse, mm^2^, *n* = 5 mice per group) in WT mice (control), WT mice receiving DSS (DSS, 3% in drinking water for 7 consecutive days) as well as mice receiving *L. reuteri* R2LC was given daily to WT mice for 14 days starting 7 days prior to DSS-treatment (*L. reuteri*-DSS). Histological analysis of the PPs height (μm, serially sectioned with all slides per PP analyzed, *n* = 5 mice per group, scale bars equal 200 μm). **b** Flow cytometry of live CD3^-^CD19^+^B220^+^ B cells, small-pre-GC-like and large-GC-like B cells in PPs (*n* = 6–17 mice per group). **c** Body weight change and disease activity index of DSS-treated mice and mice co-treated with *L. reuteri* (*n* = 9 mice per group) or FTY720 (*n* = 6 mice per group). **d** Pearson correlation of PPs B cell number and body weight loss (%) (*n* = 24 mice). **e** Bar graph depicts bacterial community composition of individual mouse, *n* = 4 mice per group. **f**–**h** Relative abundance of *Erysipelotrichaceae*, *S24-7* and *Lactobacillaceae* in the ileal microbiota (*n* = 4–9 mice per group). Data are presented as median values. Contrast analysis demonstrated that bacterial taxa changed significantly with DSS-treatment (diseased *P* < 0.05) and was preserved by *L. reuteri*-treatment (protected *P* < 0.05). Data are presented as mean ± SEM. *p < 0.05, **p < 0.01, ***p < 0.001, ****p < 0.0001 using ANOVA with Tukey’s post hoc test

As previously demonstrated [8], DSS induces colitis and profound inflammation in the colon (Additional file 1: Fig. S5A, B). Here, we found that it also severely damaged the ileal mucosa (Additional file 1: Fig. S5C) and reduced the number of CD3^-^CD19^+^B220^+^ B cells in the PPs by more than 50%. This reduction was due to decreased numbers of both pre-GC-like B cells and GC-like B cells, resulting in reduced PPs size (Fig. 5a, b). Colitis induced significant B cell accumulation in MLNs (Additional file 1: Fig. S5D) and caused ileal mucosal infiltration of IgA^+^ plasma cells (Additional file 1: Fig. S5E), but did not affect the proportion of IgA^+^ bacteria in the ileal flora (Additional file 1: Fig. S5F). In contrast, a significant increase of the level of IgA bound per bacteria (IgA^low^) was detected in DSS-treated mice when compared to control (Additional file 1: Fig. S5F). Prophylactic treatment of *L. reuteri* for 14 days prevented DSS-induced ileal disruption (Additional file 1: Fig. S5C), and delayed and attenuated colitis symptoms (Fig. 5c, Additional file 1: Fig. S5A, B). Disease activity index (DAI) [8] revealed that the protective effect was most obvious for R2LC followed by 4659, but not observed with 6475 (Additional file 1: Fig. S5G). *L. reuteri* R2LC_ΔADO-treatment also lowered the DAI compared to DSS only, demonstrating that the disease-ameliorating effects by R2LC are not mediated through adenosine signaling.

*L. reuteri*-treatment given to DSS-challenged mice preserved their PPs integrity, the number of both subsets of PPs B cells (Fig. 5a, b), and reduced the B cell accumulation in MLNs (Additional file 1: Fig. S5D). Further, *L. reuteri*-treatment reduced the ileal infiltration of IgA^+^ plasma cells (Additional file 1: Fig. S5E), and restored the DSS-induced shift of SIgA-coated bacteria (Additional file 1: Fig. S5F). A strong negative correlation between body weight loss and B cell numbers in PPs following DSS-treatment was detected (Fig. 5d), supporting that the effects by *L. reuteri* on PPs B cells are important for preventing colitis. Again, part of the probiotic effect can be explained by the local S1P/S1PR1 signaling in the PPs, as co-treatment with FTY720 to DSS-treated mice mimicked the effect of probiotics with maintained pre-GC-like B cells in PPs (Fig. 5b) and delayed disease onset (Fig. 5c, Additional file 1: Fig. S5H).

DSS-treatment induced a major shift of the bacterial community composition into a dysbiotic state (Fig. 5e), including reduced α-diversity in both ileum and colon (Fig. 4e, Additional file 1: Fig. S4C). The bacterial taxa *Erysipelotrichaceae* in the ileum (Fig. 5f) and *Escherichia-Shigella* (affiliated with *Enterobacteriaceae*) in the colon (Additional file 1: Fig. S5I) were identified as the main pathobionts, with their relative abundance increasing from less than 2% in healthy animals to up to 80% in DSS-treated mice, while the relative abundance of *S24-7* was decreased by DSS at both sites (Fig. 5g, Additional file 1: Fig. S5J). *L. reuteri*-treatment preserved the α-diversity of colonic microbiota, the ileal *Lactobacillaceae* from DSS challenge (Additional file 1: Fig. S4C, Fig. 5h), and inhibited the overgrowth of *Erysipelotrichaceae* and *Escherichia-Shigella* (Fig. 5f, Additional file 1: Fig. S5I). It also caused an obvious increase of *Bifidobacteriaceae* and *Coribacteriacease* in the ileum (Fig. 5e) and maintained the dominant *S24-7* population in the colon (Additional file 1: Fig. S5J).

Thus, we report that *L. reuteri*-treatment protects against intestinal inflammation by maintaining the functions of PPs, which modifies intestinal IgA production and prevents inflammation-induced microbiota dysbiosis.

## Discussion

The strategic location of the PPs facing the ileal lumen ensures continuous exposure to and sampling of antigens, resulting in intense B cells activation and IgA induction [10, 14, 15]. Here we demonstrate that orally administered *L. reuteri* R2LC regulates distinct B cell subsets in the PPs of mice, which ultimately results in augmented IgA production, modified microbiota, and protection against colitis. Thus, the PPs act as relays that sense, enhance and transmit signals from *L. reuteri*, which for the first time explains how gut homeostasis and the intestinal microbiome can be affected even though the probiotics represent only a small part of the microbiota while transiting.

The antibody-mediated immune-selection of gut microbiota is orchestrated by B cells in PPs that produce high or low affinity IgA in a T cell-dependent or independent manner [26]. To efficiently mount antibody responses, B cells display high transcriptional, metabolic, and functional plasticity [20, 22], making the identification based on surface markers challenging [27]. The current study therefore separated B cells in PPs by size, which resulted in phenotypically, transcriptionally and spatially distinct B cell subsets: large GC-like B cells (GL7^+^/S1PR1^−^/Ki67^+^/*Bcl6, CD69* expressing) located in the GC area, and small pre-GC-like B cells (GL7^-^/S1PR1^+^/*Bcl6*, *CCR6* expressing) of the SED in PPs.

Small pre-GC-like B cells are characterized by an innate-like phenotype not previously demonstrated for B cells in the PPs. In line with this observation, marginal zone B cells, IL7R^hi^CCR6^+^ lymphocytes, and NK-like B cells have been detected in spleen and lymph nodes [28–30], and are characterized by the production of antibacterial agents, high capacity for chemotaxis, and lipid antigen binding. Interestingly, the former two cell types are activated and mobilized using S1PR1 within lymphoid tissues for bacterial antigen sampling [28, 30]. We found that S1PR1 was exclusively expressed by the pre-GC-like B cells of the SED  and  interspersed between the FAEs, similarly to what was found in a model of oral immunization as GL7^-^CCR6^+^ B cells made close contact with M cells and antigens [14]. Komban and co-workers then speculated that these B cells thereafter migrate to GC using CCR6, thus enabling a strong antigen-specific IgA response [14]. The model of oral immunization is different from the probiotic treatment of the current study, and whether pre-GC-like B cells from the SED can become GC-like B cells, or if they are committed to defense, remains to be determined. However, we found that *L. reuteri* R2LC favors the retention of S1PR1^+^ pre-GC-like B cells in the SED area by increasing local S1P availability through different means [31]. During inflammation, the importance of S1PR1 in regulating T cells egress from lymph nodes and consecutive infiltration to the effector sites, has been intensively studied using the immunosuppressive drug FTY720 (a S1PR1 agonist) [31–34], believed to exert its effect at least partly by reducing T cell velocity in lymph nodes [35]. Here, we demonstrate that the probiotic *L. reuteri* R2LC reproduces the anti-inflammatory properties of FTY720 in DSS-colitis by affecting the population of S1PR^+^ 1 B cells in the PPs.

The largest production of IgA occurs in the intestine, where it coats 40–80% of small intestinal bacteria in human and mice and is crucial for microbiota homeostasis [36]. Naturally occurring variations in fecal IgA levels have been demonstrated, and mice with lower IgA concentration are more susceptible to infection and inflammation [9, 11], while mice with higher IgA concentration were more resistant to colitis [11]. The intestinal resident flora undergoes IgA-mediated selection, where high affinity, T cell-dependent, IgA production tends to target bacteria with pathogenic potential [26], even though the underlying mechanisms, which are probably numerous and not mutually exclusive, are still not fully understood [26]. In addition to expanding the population and the innate-like properties of the pre-GC-like B cells in the PPs, *L. reuteri*-treatment also expanded the population of large PPs B cells of the GC-like phenotype. This resulted in the induction of high affinity IgA production, as their cell cycle and proliferation gene programs were down-regulated, while T cell interactions were upregulated through the PD-1 pathway. These *L. reuteri*-primed PPs B cells resemble the Bcl6^low^CD69^hi^ post-GC B cells present in lymph nodes, which are reported to be distinct from the conventional GC B cells as they feature low proliferative but high CD40-Tfh-interactive capacity and is destined for IgA plasma differentiation [21]. *L. reuteri* also induced increased levels of local IL-21 in the PPs, which was suggested to act in synergy with TGFβ1 [37] and therefore may promote the PPs GC-like B cell-IgA differentiation via TGFβ-1 signaling. The observed effects by *L. reuteri* on augmented IgA induction and production resulting in higher titer of free IgA in the ileum and improved IgA-bacteria binding capacity, are likely to affect the composition of the gut microbiota. Indeed, the current study observes changes in the microbiome signature in the intestine of healthy as well as colitic mice. We therefore suggest that the intestinal microenvironment shaped by *L. reuteri-*induced IgA favors symbiotic bacteria growth while resists colonization of pathobionts [2] and thereby increases the resistance to DSS-induced dysbiosis.

Interactions between *L. reuteri* and the intestinal immune cells have previously been shown to influence host health and disease [7]. However, the relevance of the scientific observations for therapeutic use materializes with the delineation of the exact mechanism of actions. For instance, the probiotic *L. reuteri* strains WU and 100-23 have been demonstrated to drive the differentiation of ileal CD4^+^CD8aa^+^ intraepithelial lymphocytes that are responsible for oral tolerance [4]. In addition, *L. reuteri* DSM 17938 has been reported to shift the Th1/Th17 paradigm in a mouse model of multiple sclerosis [38]. In immune checkpoint blockade-associated colitis, *L. reuteri* ATCC PTA 6475 was found to ameliorate inflammation by inhibiting Group 3 innate lymphoid cell activities [3]. These findings underscore the diverse effects by which probiotic *L. reuteri* provide health benefit [7]. Here we add to this by demonstrating that *L. reuteri*-treatment primes the intrinsic functions of PPs B cells in healthy mice, resulting in increased IgA production and a microbiota favoring symbiotic bacteria growth while resisting colonization of pathobionts [2], and increasing the resistance to DSS-induced dysbiosis. The recent successes in the treatment of recurrences of *Clostridium difficile* infection by first- and new-in-class investigational microbiome-based products (RBX2660; NCT03244644 and SER-109; NCT03183128) evaluated for Biologics License by the FDA, are milestones to the entire field. The emerging pipeline of the therapeutic use of live bacterial products (LBPs) and microbiome therapeutics include single strains, consortia, diagnostics as well as genetically modified single strains, addressing a wide range of unmet medical needs covering skin, GI tract, lung, women’s health, oncology, and neurodegenerative disease in both human and veterinary medicine [39].

## Conclusions

In summary, we demonstrate that *L. reuteri* R2LC regulates the inter-dependent and site-specific B cell responses in PPs. We found that IgD^+^/GL7^-^/S1PR1^+^/*Bcl6, CCR6* expressing pre-GC-like B cells in the SED interact with FAE and exhibit innate-like properties, and  that *L. reuteri* upregulates the bacterial defense gene program of these cells and expands the population size in a S1P/S1PR1-dependent manner. In addition, we found that *L. reuteri* increases the population size of GL7^+^/S1PR1^-^/Ki67^+^ B cells, the effector cells in the GC area of PPs, and reprograms their gene signature to promote metabolism and GC functions. Finally, *L. reuteri* increased both TGFβ1 and Tfh-PD-1 signaling in GC-like B cells, and thereby augmented the intestinal B cell-IgA responses. Together, these results reveal that *L. reuteri* R2LC exploits the PPs B cell subsets and primes their intrinsic functions, which ultimately results in an inflammation-resistant microenvironment and a bacterial community resilient to dysbiosis in both the ileum and colon. The finding that probiotic bacteria such as *L. reuteri* utilizes the PPs to transmit and enhance their effects for the first time explains how these bacteria can confer intestinal health effects despite that they do not colonize and only constitute a small part of the total microbiota.

## Methods

### Mice

Mice used in this study were from a C57Bl/6 background with wild type or CX3CR1^GFP/+^ genotype. WT male mice were purchased from Taconic M&B, Denmark. Mice were housed under standardized conditions (21–22 °C, 12-h light and 12-h dark cycle) in SPF facility and were used at 10–12 weeks old under guidelines of the Swedish National Board for Laboratory approved by the Swedish Laboratory Animal Ethical Committee in Uppsala.

### Bacteria preparation and biologics

*Limosilactobacillus reuteri* subsp. *rodentium* R2LC (a kind gift from Siv Ahrné, Lund University, Sweden) [40], *L. reuteri* subsp. *reuteri* ATCC PTA 4659 and *L. reuteri* subsp. *reuteri* ATCC PTA 6475 (kind gifts from BioGaia AB, Sweden) [41] were grown in MRS broth (Oxoid) at 37 °C for 20 h. Bacteria were harvested at early stationary phase, washed with PBS, concentrated 100X and stored at -80 °C in freezing solution (0.82 g K_2_HPO_4_, 0.18 g KH_2_PO_4_, 0.59 g sodium citrate, 0.25 g MgSO_4_ × 7 H_2_O, and 172 mL glycerol (87%) diluted to 1000 mL with dH_2_O). 10^8^ cfu (resuspended in 100 μl of PBS) were given perorally (*p.o.*) for 7 consecutive days. For PD-1 blocking experiments, mice were injected *i.p.* with 200 μg α-PD-1 (RMP1-14, BioXcell) or equivalent rat IgG (2A3, BioXcell) every other day during the 7-day course of *L. reuteri*-treatment. For CD4^+^ T cell depletion, 200 μg of rat-anti mouse CD4 mAb (GK1.5, BioXcell) were injected *i.p*. at days 3 and 6, as illustrated (Fig. 4i). DSS-mediated colitis was induced by administration of 3% (w/v) DSS (M.W. = 40,000 Da; TdB Consultancy AB) in drinking water for 7 consecutive days. The onset of colitis and severity was assessed daily based on clinical measurements including weight loss, stool consistency, and blood reaction in the feces, calculated as a disease activity index scoring from 0 to 4 [8]. *L. reuteri* R2LC was given daily for 14 days starting 7 days prior to DSS-treatment (*L. reuteri*-DSS). In addition, mice were subjected to *i.p.* injection of a S1PR1 agonist FTY720 (1 mg/kg body weight) or a vehicle control for 4 consecutive days during DSS and sacrificed 3 days later.

### Plasmid generation and construction of *L. reuteri* R2LC_∆ADO

The draft genome of *L. reuteri* R2LC was used to identify the gene putatively encoding the 5′-nucleotidase (locus tag RMX24361) [42]. The upstream (910 base pairs) and downstream (945 base pairs) regions with oligonucleotide pairs oVPL3303-oVPL3304 and oVPL3305-oVPL3306 were then amplified by PCR, respectively (Additional file 1: Table S3). With oligonucleotide pair oVPL187-oVPL188, the backbone of plasmid pVPL3002 (a suicide vector) was amplified and was integrated in the chromosome, which renders cells sensitive to vancomycin [43]. The three PCR amplicons were fused by ligation cycle reaction [44] with bridging oligonucleotides oVPL3307, oVPL3308, and oVPL3309, followed by ethanol precipitation of the DNA [45], and was visualized by Pellet Paint co-precipitant (EMD Millipore). Electrocompetent *E. coli* EC101(encodes *RepA in trans* to support replication of *repA*^−^ plasmids) were prepared using standard procedures [45, 46]. The insertion of the 1.8 kb fragment was confirmed by PCR (oligonucleotide pair oVPL49-oVPL97, which flanks the cloning site in pVPL3002, Additional file 1: Table S3). While the integrity of the cloned DNA sequence was confirmed by sequence analysis with the Sanger method.

Next, we generated an in-frame deletion of *L. reuteri* RMX24361 according to a previously established protocol [43] and constructed *L. reuteri* R2LC_∆ADO. Briefly, we electroporated 5 μg of the pVPL3002-derivative containing the deletion cassette into *L. reuteri* R2LC. Following recovery, cells were plated on MRS agar containing 5 μg/ml erythromycin. Colonies were screened by PCR using oligonucleotide combination oVPL3310-oVPL3311-oVPL97 and oVPL3310-oVPL3311-oVPL49 to identify homologous recombination upstream or downstream, respectively, of the target gene (Additional file 1: Table S3). A single-crossover mutant was cultured for 10 generations and recovered on MRS plates containing 400 μg/ml vancomycin. Colonies resistant to vancomycin have lost the suicide vector following a second homologous recombination event to yield either a wild-type or recombinant genotype. Finally, the deletion genotype was confirmed by PCR with oligonucleotide oVPL3310-oVPL3311 and by sequence analysis with the Sanger method.

### Histology

The terminal Peyer’s patches, distal ileum, and colon tissues were fixed in 4% paraformaldehyde (PFA) overnight, dehydrated in 70% ethanol, and embedded in paraffin. Four μm thick sections were stained with hematoxylin and eosin (H&E). The whole terminal PP was serially sectioned. All slides of one PP were evaluated, and the arch of PP’s dome was identified, thus the height was measured. Ileal tissue sections were scored (blindly) by assessing mucosal architecture (villus blunt, distortion, and atrophy), epithelial changes (erosion and ulceration), and inflammatory immune cell infiltration based on a 0-12 scoring system (Additional file 1: Table S4). Duplicate was used for all tissue sections and was imaged with a light microscope (Leica DFC 420C).

### Immunofluorescent microscopy

Peyer’s patches, distal ileal and distal colon tissue samples were fixed in 4% PFA overnight, dehydrated in 30% of sucrose, embedded in O.C.T. (Tissue-Tek), and sectioned (10 μm). Sections were fixed in either 4% PFA or acetone shortly for 2 minutes and permeabilized (0.5% Triton™ X-100, Sigma-Aldrich) before antibody incubation. Antibodies raised against the following mouse antigens were used: B220 (AF647, cloneRA3-6B2, BioLegend), CD3 (AF488, clone17A2, BioLegend), CD21/CD35 (AF594, clone7E9, BioLegend), CD138 (unconjugated, clone281-2, BioLegend), CD279/PD-1 (AF647, clone29F.1A12, BioLegend), GL7 (FITC, cloneGL7, BD Bioscience), IgA (FITC, cloneC10-3, BD Bioscience), IgD (Pacific blue, 11-26c2a, BioLegend), Ki67 (unconjugated, cloneMIB-1, Abcam), DEFa3 (unconjugated, RBM6, antibodies-online), pimonidazole (Pacific Blue, Hypoxyprobe), and Phalloidin (AF555). Detection of CD138, Ki67, and DEFa3 required secondary antibodies to amplify signals with goat anti-rat-IgG-AF555 and chicken anti-rabbit-IgG-AF488 (Thermo Fisher Scientific), respectively. Nucleus was stained with Hoechst 33342 (Thermo Fisher Scientific) and mounted using Fluoromount-G® (SouthernBiotech). For whole mount staining, PPs from CX3CR1^GFP/+^ mice were stained with intravenous CD31 (AF555 conjugated, clone309, eBioscience) prior to sampling, then PPs tissue was removed and fixed using BD Cytofix/Cytoperm solution for 1 hr and permeabilized with BD Perm/Wash buffer for 5 min before antibody incubation. The whole PPs were stained with wheat germ agglutinin (CF®405 M, Biotium) and placed in a 30% solution of glycerol in PBS on the slide. All samples were imaged with a Zeiss confocal Laser Scanning Microscope 780.

### Flow cytometry and cell sorting

All visible PPs were harvested and pooled when preparing single cell suspensions for flow cytometry. Single-cell suspensions from PPs, MLNs, and spleen were prepared by mashing organs through 40 μm cell strainers in PBS containing 0.05% FBS and 2 mM EDTA (MLNs or spleen cells were used as staining control). PPs cells were stained with anti-CD3 biotinylated antibody (17A2, BioLegend) and were subjected to Magnetic Cell Sorting system (Miltenyi Biotec) to separate CD3^+^ and CD3^-^ fractions. Samples were blocked with anti-CD16/32 (2.4G2, BD Bioscience), stained with antibodies anti- B220 (RA3-6B2), CD19 (6D5), CD3 (17A2), CD4 (GK1.5), CD279 (29F.1A12), NK1.1 (PKB6) from BioLegend; CXCR5 (2G8), GL7 (GL7); S1P1/EDG-1 (T4-H28) from R&D; CD8α (5H10), TCRαβ (H57-597) from Thermo Fisher Scientific; and CD11c (N418), CD62L (MEL-14) from eBioscience. Dead cells were excluded from analysis using the Live/Dead Aqua Viability Kit (Thermo Fisher Scientific) or 7AAD (BD Bioscience). For intracellular staining of HIF-1α (351-400/826, Bioss), TGFβ1 (860206, R&D), IL-21 (149204, R&D), and DEFa3 (RBM6, antibodies-online), cells were fixed and permeabilized using either Foxp3/Transcription Factor Staining Buffer Set (eBioscience) or Fixation/Permeabilization Solution Kit (BD Cytofix/Cytoperm) according to the manufacturer’s instructions (BD Bioscience).

For B cell sorting, single cell suspension of PPs was prepared and was enriched by negative selection using MACS columns with a Cocktail of biotin-conjugated monoclonal antibodies following the manufacturer’s instructions (Miltenyi Biotech). The live lineage negative cells (Lin^-^, *i.e.* CD3ε^-^, CD4^-^, CD8a^-^, CD49b^-^, Gr-1^-^ and Ter119^-^) were stained with anti-CD19 and anti-B220 antibodies and sorted by FSC-A as small B and large B with FACS-AriaIII (BD Bioscience).

### In vivo proliferation assay

Mice were injected *i. p.* with 500 μg of the nucleoside analog EdU (Baseclick GmbH) in PBS twice, i.e., 24 h and 3 h before euthanizing. After, PPs were prepared for single cell suspension and stained for surface antigens as described, followed by EdU detection using the EdU FC Kit 647 (Baseclick GmbH) according to the manufacturer’s protocol, with anti-Ki67(SolA15, eBioscience) staining afterwards.

### RNA extraction and microarray analysis

Total RNA of sorted small B and large B cells (live Lin^−^CD19^+^B220^+^) and PPs tissue were extracted with RNeasy Plus Micro Kit (QIAGEN). Total RNA of sorted B cells was submitted to Array and Analysis Facility, Department of Medical Science, Uppsala University. RNA integrity was evaluated by Agilent 2100 Bioanalyzer system (Agilent Technologies). Biotinylated sense-strand cDNA from the entire expressed genome was generated according to the GeneChip™ WT Pico Reagent Kit Manual (P/N 703262, ThermoFisher Scientific). Clariom™ D mouse arrays (previous name: GeneChip™ Mouse Transcriptome Array 1.0) were hybridized for 16 hours in a 45 °C incubator, rotated at 60 rpm. According to the GeneChip™ Expression Wash, Stain and Scan Manual (P/N 702731, Thermo Fisher Scientific) the arrays were then washed and stained using the GeneChip™ Fluidics Station 450 and finally scanned using the GeneChip™ Scanner 3000 7G.

The raw data were normalized to gene level in Expression Console (Affymetrix) using the SST-RMA version of the robust multi-array average method. Subsequent analysis of the gene expression data (log_2_ expression of genes ranging between 0 and 20) was carried out in the statistical computing language R. In order to search for the differentially expressed genes between groups, an empirical Bayes moderated t-test was applied using the ‘limma’ package (version 3.26.1), p-values were adjusted with false discovery rate control (FDR) using the method of Benjamini and Hochberg. Genes differentially expressed were further selected and put into The Database for Annotation, Visualization and Integrated Discovery (DAVID) v6.8 for gene enrichment ontology analysis related to Biological Process (BP). That is, expression values under 5 were considered background. For large B cells and small B cells comparison, genes whose fold-change over 1 and FDR *q* < 0.05 (total 383 gene transcripts). For *L. reuteri*-treated large B and control large B, FDR *q* < 0.05 (total 285 gene transcripts) and for *L. reuteri*-treated small B and control small B, FC over 1 and FDR *q* < 0.1 (26 gene transcripts).

### Quantitative real-time PCR

Total RNA was transcribed with a High-Capacity cDNA kit (Thermo Fisher Scientific). Quantitative RT-PCR was performed using Fast SYBR™ Green Master Mix on a QuantStudio™5 Real-Time PCR System, in a 384-well format (Thermo Fisher Scientific). The geometric mean of 3 reference genes *GAPDH*, *TBP*, and *36B4* was used for normalization. Relative expression of target genes was determined. The specific primers are listed in Additional file 1: Table S3.

### Analysis of IgA-bound bacteria and free IgA

IgA binding bacteria were analyzed by flow cytometry as previously described [47, 48]. In brief, luminal content from the ileum and colon were collected, suspended in filtered PBS (100 μl to 10 mg feces), homogenized, and centrifuged at 400*g* for 5 min to remove large debris. Supernatant-containing bacteria were filtered through a sterile 70 μm strainer and centrifuged at 8000*g* for 10 min to separate free immunoglobulins. The supernatant was further centrifuged at 19,000*g* for 10 min and collected for free IgA assay using IgA ELISA quantitation Set (Bethyl). The pellet was resuspended in 1 ml of BSA-PBS (1% w/v) and blocked on ice for 15 min. After being stained with anti-mouse IgA (FITC, C10-3, BD Bioscience) for 30 min on ice, samples were washed with filtered PBS, resuspended in 4 μg/ml propidium iodide (PI)/PBS, and analyzed by LSR Fortessa (BD Bioscience).

### Multi-plex mesoscale assay and ELISA

IL-10, IFN-γ, TNF-α, IL1-β, IL-6, CXCL1, IL-2, IL-4, IL-5, and IL-12 were measured in PPs, distal colon using Multi-Plex Mesoscale assay following manufacturer’s instructions (MesoScale Discovery). Tissues were homogenized and measured for protein concentration by the BCA method and values were normalized to tissue protein content. TGFβ1 was analyzed by ELISA (Mouse TGF-beta 1 DuoSet, R&D System), serum IgG and IgA concentrations were determined by ELISA quantitation Set (Bethyl).

### DNA extraction and microbiota analysis

DNA was extracted from 180 to 220 mg of intestinal lumen content using QIAamp Fast DNA Stool Mini kit (Qiagen). The Illumina MiSeq Dual Index Amplicon Sequencing was performed. Briefly, the bacterial V3-V4 16S rRNA gene regions were PCR amplified from each sample using a composite forward primer and a reverse primer containing a unique 8-base index primer, designed to tag PCR product from respective samples. The forward primer was Illumina adapter-N4-341F: 3′-ACACTCTTTCCCTACACGACGCTCTTCCGATCTNNNNCCTACGGGNGGCWGCAG-5′. The reverse primer was Illumina adapter-805R:3′-AGACGTGTGCTCTTCCGATCTGACTACHVGGGTATCTAATCC-5′. PCR reactions consisted of master mix (0.5 μl of each primer, 4 μl of Q5 reaction buffer, 0.2 μl (0.02 U/μl) Q5 HF DNA polymerase (New England Biolabs), 2 μl of dNTPs, 11.8 μl of Milli-Q water) and 1 μl of DNA template. Reaction conditions were 1 min at 98 °C, followed by 20 cycles of 10 s at 98 °C, 30 s at 58 °C and 30 s at 72 °C, and a final extension step at 72 °C for 2 min on a Bio-Rad thermocycler. Duplicates were run in 20 μl reactions for each sample, combined, purified with Agencourt Ampure magnetic purification beads (Beckman Coulter). Products were visualized by gel electrophoresis. Then, a second PCR was conducted using 1:10 dilutions of the first PCR product for attaching standard Illumina handles and index primers. The steps were the same as mentioned above, except that it was 18 thermal cycles and the annealing temperature was 63 °C. PCR products were quantified with Picogreen dsDNA assay according to the manufacturer’s instruction (Thermo Fisher Scientific) to facilitate pooling equimolar amounts of amplicon for sequencing. Finally, a master DNA pool was generated and sequenced on an Illumina HiSeq 2500 sequencer at the SciLifeLab SNP/SEQ sequencing facility (Uppsala, Sweden).

The Illumina sequencing data output was processed according to the cut-offs and pipeline developed by Sinclair et al. [49]. Sequences were assigned to operational taxonomic units (OTUs) by using a closed reference-based OTU picking method in QIIME v1.8. For every OTU, the sequence was checked as a query against the SILVAMOD database using the CREST software version 2.0. For comparative measurements of total bacterial load, a primer for total 16S rRNA primers was used. The data were normalized by sample weight.

### Statistical analysis

Statistical analysis was performed using Graphpad Prism software v.6.0. Two-tailed Student’s *t* test was used for direct comparison of two groups, and one-way analysis of variance (ANOVA) with Tukey’s post hoc test to compare all groups. Difference of DAI was calculated and compared as area under the curve. For microbial community composition analysis, bioinformatics was performed using the cumulative-sum scaling method [50]. VEGAN package version 2.0-7 and the R statistical framework version 2.11 were used to perform multivariate analysis of microbiota, α-diversity is represented by Shannon index and ANOVA analysis with Contrast. Data were presented as mean ± SEM., *p* < 0.05 was considered significant.

## Supplementary Information

**Additional file 1: Fig. S1.** The staining and gating strategy of cell subsets isolated from Peyer’s patches and *in vivo* imaging of whole tissues. **Fig. S2.** Description of B cell identity and features of large B and small B cells in PPs. **Fig. S3.** Changes of lymphocyte populations in response to *Limosilactobacillus reuteri* strains and FTY720-treatment. **Fig. S4.** Effects of *L. reuteri* strains on B cell-IgA, colonic microbiota and T cell responses. **Fig. S5.** Effects of *L. reuteri* and/or DSS on the intestine. **Table S1.** Enrichment of gene ontology categories in large B cells versus small B cells. **Table S2.** Gene ontology enrichment score (functional cluster analysis) of highly repressed genes by *L. reuteri*-treatment in large-B compared to large-B cells from control. **Table S3.** Primers used for quantitative-RT-PCR and oligonucleotides. **Table S4.** Histological scoring system for DSS induced ileal disruption.

**Additional file 2: Movie S1.** Representative 3D image (60 μm thick z stack) of intravital microscopy showing Peyer’s patches microanatomy *i.e.* lining beneath FAE (WGA, white) is the SED region occupied by antigen presenting cells (CX3CR1^GFP/+^, green) and abundant B cell population (B220^+^, magenta), where a dense vascular network is identified (CD31^+^, red).

**Additional file 3: Movie S2.** Representative 3D image (60 μm thick z stack) of whole mount stained PPs from intravital microscopy studies. B cells (B220^+^, magenta) interspersed in FAE (wheat germ agglutinin, white) are enumerated and the cell area is measured.

## Data Availability

The microarray data generated during this study are available at the NCBI Gene Expression Omnibus (GEO) database under accession number: GSE114571. The bacterial 16S rRNA amplicon sequence data generated during this study are available in European Nucleotide Archive database under accession number: PRJEB12149 (ERP013591).

## Abbreviations

AICDA: Activation-induced cytidine deaminase
BP: Biological process
DAI: Disease activity index
DCs: Dendritic cells
DSS: Dextran sodium sulfate
EdU: 5-ethynyl-2′-deoxyuridine
FAE: Follicle-associated-epithelium
FDR: False discovery rate
GCs: Germinal centers
GI: Gastrointestinal
Gsk-3α: Glycogen synthase kinase 3
IBD: Inflammatory bowel diseases
IgA: Immunoglobulin A
LBPs: Live bacterial products
MLN: Mesenteric lymph nodes
OTUs: Operational taxonomic units
PCA: Principal component analysis
PD-1: Programmed cell death protein 1
PFA: Paraformaldehyde
PI: Propidium iodide
PPs: Peyer’s patches
SED: Sub-epithelial dome
S1P: Sphingosine-1-phosphate
Tfh: T follicular helper
TGFβ: Transforming growth factor β
