# Supplementary material for: Distinct B cell subsets in Peyer’s patches convey probiotic effects by Limosilactobacillus reuteri
Source: Microbiome. 2021 Oct 3;9:198. doi: 10.1186/s40168-021-01128-4 (PMC8487498; doi:10.1186/s40168-021-01128-4)
Supplement: Supplementary file 2 — Additional file 1: Fig. S1. The staining and gating strategy of cell subsets isolated from Peyer’s patches and in vivo imaging of whole tissues. Fig. S2. Description of B cell identity and features of large B and small B cells in PPs. Fig. S3. Changes of lymphocyte populations in response to Limosilactobacillus reuteri strains and FTY720-treatment. Fig. S4. Effects of L. reuteri strains on B cell-IgA, colonic microbiota and T cell responses. Fig. S5. Effects of L. reuteri and/or DSS on the intestine. Table S1. Enrichment of gene ontology categories in large B cells versus small B cells. Table S2. Gene ontology enrichment score (functional cluster analysis) of highly repressed genes by L. reuteri-treatment in large-B compared to large-B cells from control. Table S3. Primers used for quantitative-RT-PCR and oligonucleotides. Table S4. Histological scoring system for DSS induced ileal disruption. [file 40168_2021_1128_MOESM2_ESM.docx]

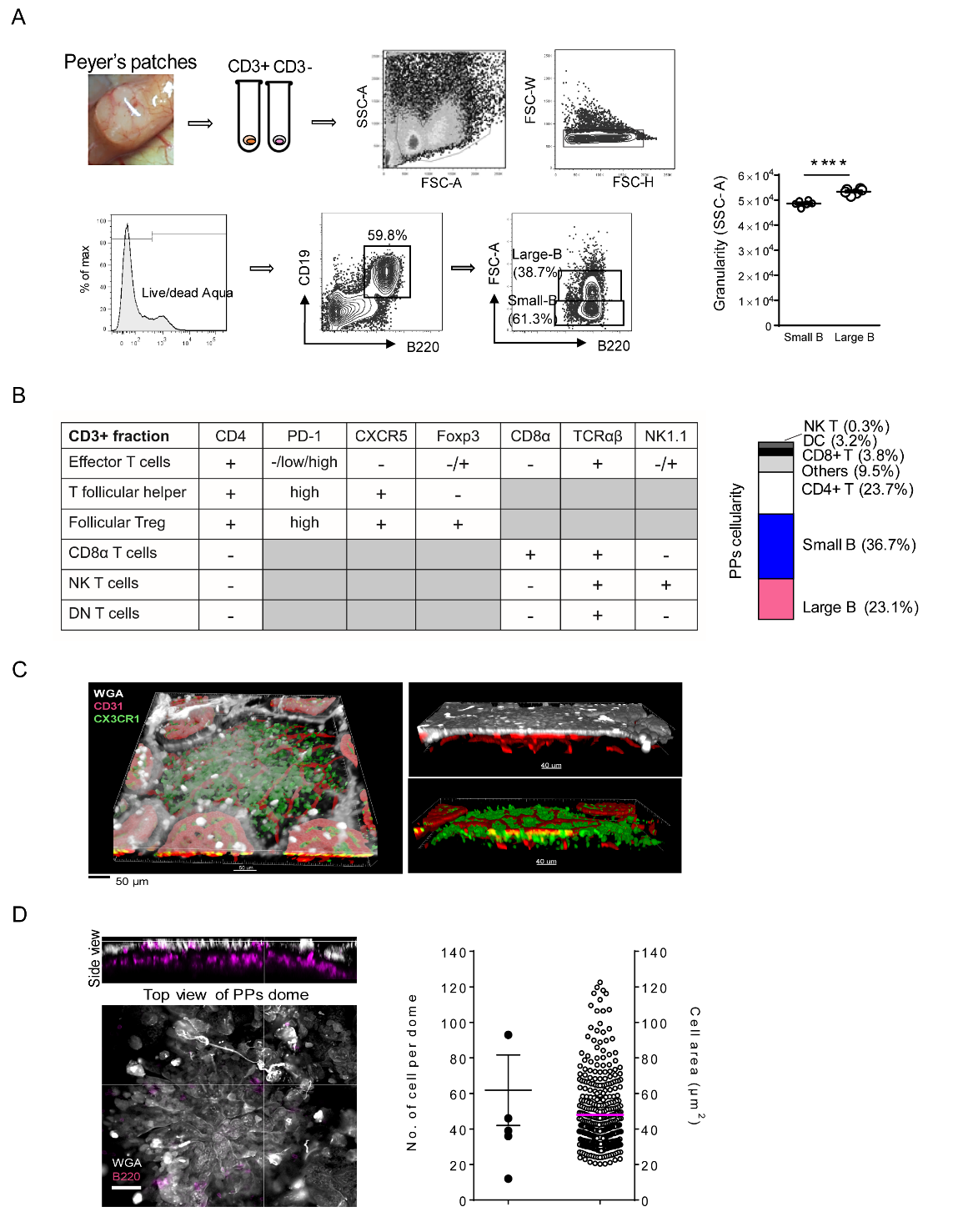
**Fig. S1** The staining and gating strategy of cell subsets isolated from Peyer’s patches and *in vivo* imaging of whole tissues. **A** All visible PPs were harvested and pooled when preparing single cell suspensions for flow cytometry. Cells were separated and stained with different surface markers. Upon flow cytometry analysis, doublets were removed, and live cells were selected. The CD3^-^CD19^+^B220^+^ B cells were then gated. Side scattered light area (SSC-A) data show that the small and large B cell populations differ from each other also in granularity (*n* = 6 mice per group). **B** The CD3^+^ fraction of cells was stained and analyzed for T cell subsets as indicated in the table. DN, double negative for CD4^-^CD8α^-^. Immune cell populations distribution in PPs (DC, dendritic cell; NK, natural killer). **C** Whole PPs were stained with wheat germ agglutinin for epithelium (white) and vessels were stained (*i.v.*) with anti-CD31 (red). Representative images show top view (left) and side views (right) of PPs from CX3CR1^GFP/+^ mice (*n* = 5 mice per group). **D** Representative images of a PP dome side and top views, scale bar equals 100 µm. Number of observed B cells (anti-B220, magenta) interspersed in the epithelium (white, left axis, average of two domes per PP per mouse, *n* = 5 mice). Areas (µm2) of the FAE interspersed B cells (right axis, each dot is one cell). Data are means ± SEM. *****p < 0.0001* using two tailed Student’s t test.

**
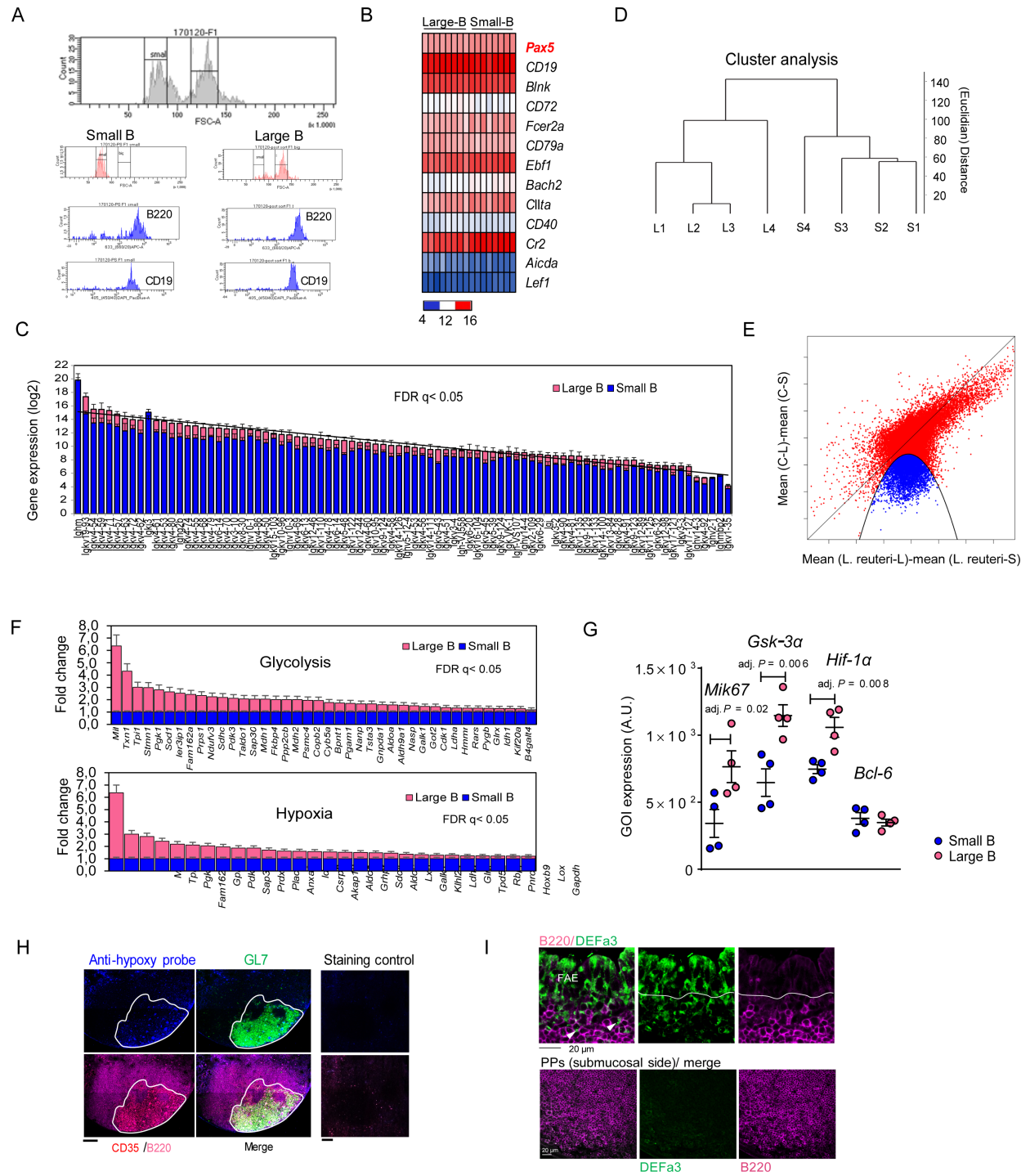
Fig. S2** Description of B cell identity and features of large B and small B cells in PPs. **A** Live Lin^-^ CD19^+^B220^+^ B cells were subjected to FACS-AriaIII and sorted by FSC-A as small B and large B cells. mRNA was extracted and purified for microarray analysis (*n* = 4 samples per group). A fraction of small B and large B were sorted back to confirm their identity (CD19^+^B220^+^). **B** B cell specific genes expression. *Pax5* is highlighted as the master transcriptional regulator that controls the identity of B lymphocytes. Each column represents one sample. **C** Expression of immunoglobulin genes of B cells (log2 transformed), mapped to Igh-V (heavy chain, variable region), Igκ/λ-V (light chain, variable region) and Igh-C (constant region) and ordered. **D, E** Cluster analysis with Euclidian distance of microarray data of small and large B cells and analysis of the pattern of their gene expression showing the similarity and the difference (*n* = 4 samples per group). **F** Genes involved in glycolysis and hypoxia that are significantly higher expressed in large B compared to small B cells (genes differentially expressed and mined in Molecular Signatures Database, MSigDB v6.1). **G** Expression of *Mki67*, *Gsk-3α*, *Hif-1α* and *Bcl-6* in large and small B cells. Data were normalized and presented as gene of interest (GOI), arbitrary unit (A.U.). **H** Representative images of anti-pimonidazole (blue) staining of PPs sections together with anti-GL7 (green), anti-B220 (magenta) and anti-CD35 (red), scale bars equal 100 µm. **I** Representative images of PPs stained with anti-B220 (magenta) and anti-DEFa3 (green). Data represent at least three independent experiments. White line segregates FAE from underlying dome, white arrows highlight DEFa3^+^ B cells. Data are means ± SEM.

**
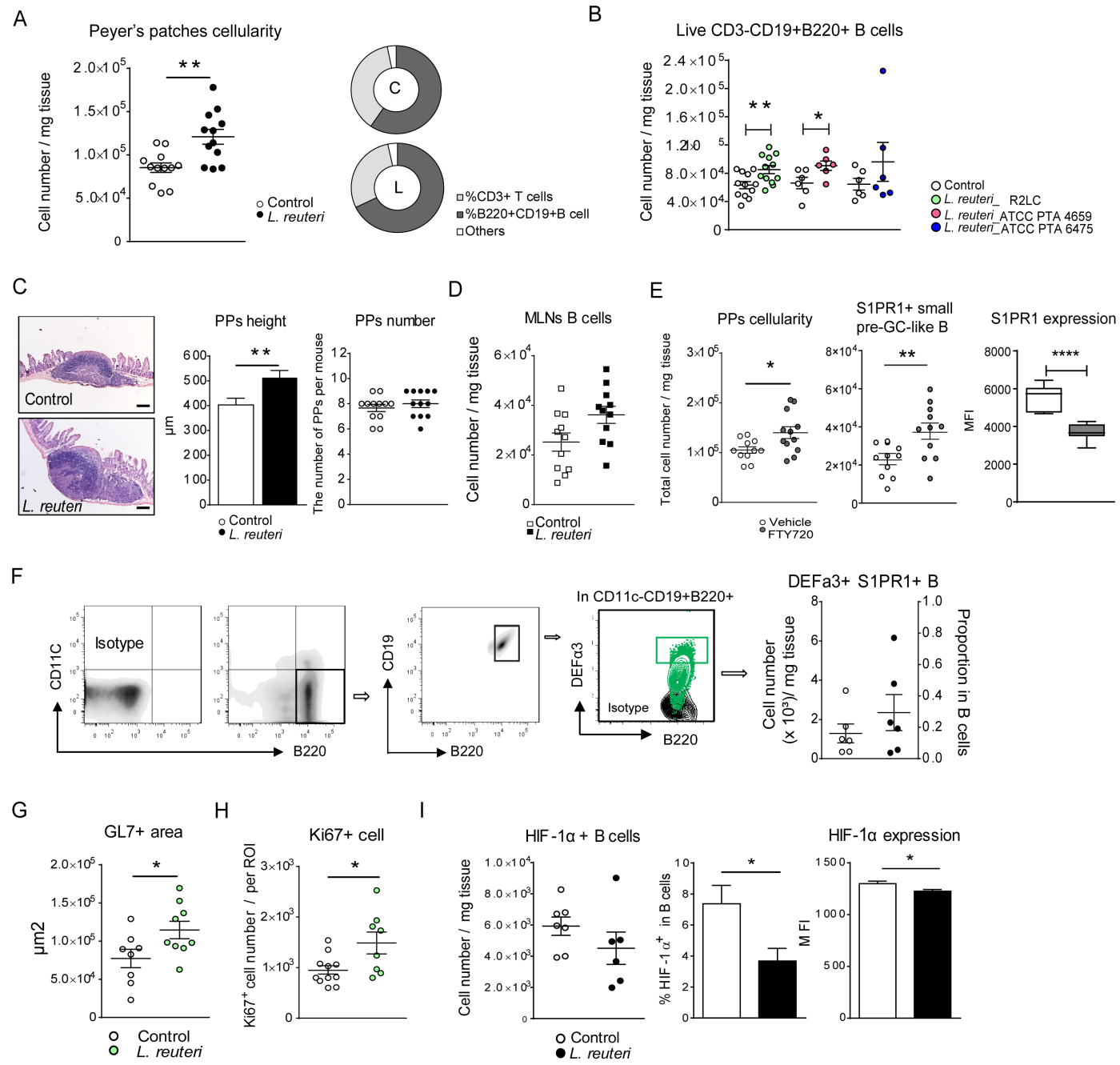
Fig. S3** Changes of lymphocyte populations in response to *Limosilactobacillus reuteri* strains and FTY720-treatment. Wild-type mice were treated with 10^8^ *L. reuteri* strains (*i.e.* *L. reuteri* R2LC, *L. reuteri* ATCC PTA 4659 or *L. reuteri* ATCC PTA 6475) per orally for 7 consecutive days comparing to control. **A** The total number of cells and relative number of lymphocytes in PPs of mice from either *L. reuteri* R2LC-treatment or control (*n* = 12 mice per group). **B** Flow cytometry quantification of the number of B cells (live CD3^-^CD19^+^B220^+^) in PPs in response to different strains of *L. reuteri* (*n* = 6-12 mice per group). **C** The height of PPs was measured (µm) from the H&E-stained complete terminal PP (serially sectioned with all slides evaluated). Scale bars equal 200 µm, *n* = 5 mice per group. The number of PPs per mouse (*n* = 12 mice per group)*.* **D** Flow cytometry analysis of the number of B cells (live CD3^-^CD19^+^B220^+^) in MLNs (*n* = 11 mice per group). **E** The numbers of total cells, S1PR1^+^ small pre-GC-like B cells in PPs of mice from either FTY720-treatment or vehicle control and S1PR1 expression (*n* = 11-12 mice per group). **F** Representative flow cytometry profiles of DEFa3^+^ B cells (live CD11C^-^CD19^+^B220^+^), the number and percentage in B cell population of PPs from control mice (*n* = 6 mice per group). **G** Quantification of GL7^+^ area with immunohistochemistry of PPs stained with anti-B220, anti-GL7 and Hoechst (related to Fig. 3a, *n* = 8-9 mice per group). **H** Quantification of Ki67^+^ cells in PPs stained with anti-Ki67, anti-B220 (related to Fig. 3c, *n* = 8-11 mice per group). **I** Flow cytometry quantification of the number of HIF-1α^+^ B cells (live CD3^-^CD19^+^B220^+^), percentage and HIF-1α expression (MFI, *n* =6-7 mice per group). Data are means ± SEM*. *p < 0.05, **p < 0.01* using two tailed Student’s t test


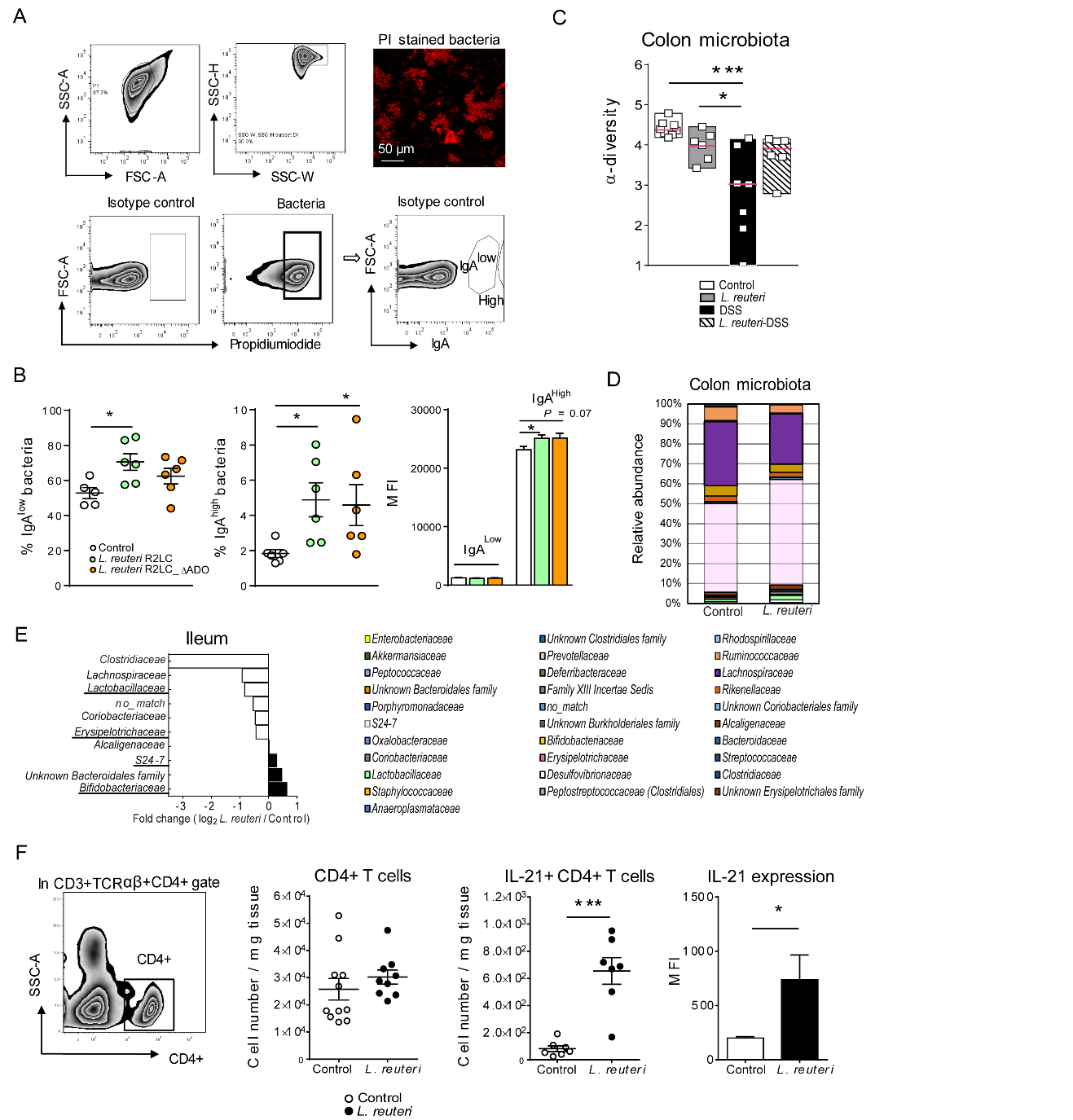
**Fig. S4** Effects of *L. reuteri* strains on B cell-IgA, colonic microbiota and T cell responses. **A** Representative flow cytometry staining of mice feces and isotype controls showing gating strategy of IgA bound bacteria and propidium iodide-stained bacteria, also evaluated by confocal microscopy. Scale bar equals 50 µm. **B** The numbers of B cells (live CD3^-^CD19^+^B220^+^), small B and large B in PPs, as well as IgA^+^ bacteria and IgA MFI in ileum were determined by flow cytometry in response to *L. reuteri* R2LC and *L. reuteri* R2LC_ΔADO comparing to control (*n* = 5-6 mice per group). **C** Colonic microbiome assessed by 16S rRNA gene amplicon sequencing. Microbial community diversity was calculated as α-diversity in mice treated with *L. reuteri* and/or DSS compared to controls, *n* = 5-9 mice in each group. Data are presented as median values. **D** Average relative abundance of bacterial taxa. **E** Average relative abundance of bacterial taxa and fold change of log2 expressed relative abundance of top 10 shifted bacterial taxa (*L. reuteri* group/control group, *n* = 4-5 mice per group). **F** Flow cytometry profile of CD4^+^ T cells (in live CD3^+^TCRαβ^+^). The number of CD4^+^ T cell in PPs (*n* = 11 mice in control and *n* = 9 mice in *L. reuteri* group) and IL-21^+^CD4^+^ T cells and IL-21 expression in PPs (*n* = 7 mice per group). Data are means ± SEM. **p < 0.05, ***p < 0.001* using ANOVA with Tukey’s post hoc test or two tailed Student’s t test

**
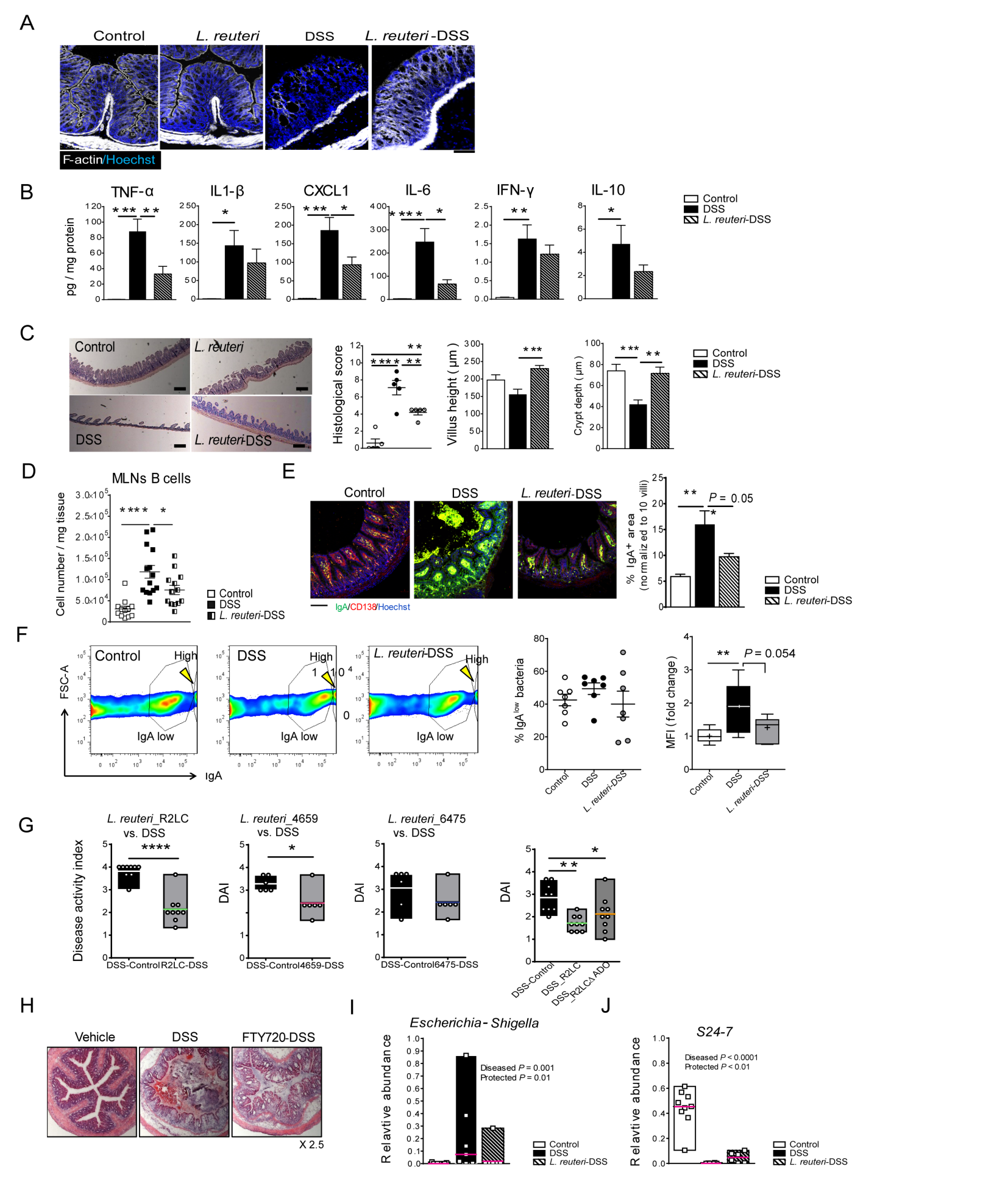
**

**Fig. S5** Effects of *L. reuteri* and/or DSS on the intestine. WT mice were administered 3% of DSS in drinking water for 7 consecutive days. 10^8^ *L. reuteri* of different strains (*L. reuteri* R2LC, *L. reuteri* R2LC_ΔADO, *L. reuteri* ATCC PTA 4659 or *L. reuteri* ATCC PTA 6475) was given daily for 14 days starting 7 days prior to DSS-treatment. Whereas FTY720 (1 mg/kg body weight) was *i.p.* injected for 4 consecutive days during DSS. **A** Immunohistochemical analysis of distal colon histomorphology. Representative images of colon sections from control mice, *L. reuteri* and/or DSS stained with Phalloidin (white) and Hoechst (blue), scale bars equal 100 µm (duplicates, *n* = 5 mice per group). **B** Colonic tissue was analyzed for cytokine/chemokine production by Multi-Plex Mesoscale assay (*n* = 6-8 mice per group). Data were normalized to tissue protein content. **C** Representative images of distal ileum of mice from control, *L. reuteri*, DSS or *L. reuteri*-DSS-treated groups (*n* = 5 mice per group), scale bars equal 200 µm. Ileal histopathology score was evaluated based on a 0-12 scoring system. Villus height and crypt depth was measured (*n* = 5 mice per group). **D** Flow cytometry analysis of the number of B cells (live CD3^-^CD19^+^B220^+^) in MLNs (*n* = 11-15 mice per group). **E** Representative staining of lamina propria IgA^+^ plasma cells in the distal ileum (anti-IgA [green], anti-CD138 [red] and Hoechst [blue]) and quantification in sectioned ileum. *n* = 5 mice per group, two slides per mouse were analyzed. Scale bar equals 100 µm. **F** Flow cytometry analysis of IgA^+^ bacteria in ileum (*n* = 7 mice per group). **G** Disease activity index was determined on the last day of experiment (*n* = 6-9 mice per group). **H** Representative images of colonic tissue section of control (vehicle) mice, DSS or FTY720-DSS-treated mice (*n* = 3 mice per group, two slides per mouse were analyzed). **I, J** Relative abundance of bacterial phylotype *Escherichia-Shigella* (*Enterobacteriaceae*) and *S24-7* in colonic microbiota, *n* = 5-9 mice per group. Data are presented as median value. Contrast analysis demonstrated that bacterial taxa changed significantly with DSS-treatment (Diseased *P* < 0.05) and preserved by *L. reuteri*-treatment (Protected *P* < 0.05). Data are means ± SEM. **p < 0.05, **p < 0.01, ***p < 0.001, ****p < 0.0001* using ANOVA with Tukey’s post hoc test.

Table S1 Enrichment of gene ontology categories in large B cells versus small B cells.

| **Term** | **Count** | **%** | **Genes highly expressed in large B compared to small B** | **Fold Enrichment** | **FDR** |
| --- | --- | --- | --- | --- | --- |
| GO:0007049~cell cycle | 66 | 75,0 | *MAEA, PTTG1, RPS3, RAD21, CASP8AP2, MIS18A, RHOA, VPS4A, NSMCE2, NUP37, CCNK, RBBP4, ZC3HC1, RAN, CCNH, LIG1, PAPD7, MCM2, CDK4, MCM3, PPP1CC, MCM4, PPP1CB, MCM5, MCM6, ARL3, STRA13, PPM1G, MAD2L1, CHMP1B, ZWINT, TBRG1, CDK2AP1, HAUS7, PSME3, MAPRE2, ARL8B, RUVBL1, MAD2L2, UBE2S, NUP43, SPAST, TRIOBP, GNAI3, 2610002M06RIK, GNAI2, ARF6, ANAPC11, CCNG1, VRK1, USP39, PAFAH1B1, FAM32A, ING1, NSUN2, NUDC, BECN1, NASP, GMNN, SYCE2, SMC2, PTP4A1, CKS2, BRE, CUL4B, SMC1A* | 22,09 | 1,9E-74 |
| GO:0051301~cell division | 51 | 58,0 | *MAEA, PTTG1, RPS3, CDC42, RAD21, MIS18A, RHOA, NSMCE2, VPS4A, NUP37, CCNK, ZC3HC1, RAN, LIG1, PAPD7, PPP1CC, CDK4, PPP1CB, MCM5, ARL3, STRA13, CHMP1B, MAD2L1, ZWINT, MAPRE2, HAUS7, ARL8B, RUVBL1, MAD2L2, UBE2S, NUP43, SPAST, TRIOBP, GNAI3, GNAI2, 2610002M06RIK, ARF6, ANAPC11, CCNG1, VRK1, USP39, PAFAH1B1, NSUN2, NUDC, BECN1, SYCE2, SMC2, CKS2, BRE, SMC1A, TXNL4A* | 28,02 | 8,3E-59 |
| GO:0007067~mitotic nuclear division | 30 | 34,1 | *TRIOBP, ANAPC11, PTTG1, CCNG1, RPS3, VRK1, RAD21, MIS18A, NSMCE2, PAFAH1B1, NUP37, NSUN2, NUDC, CCNK, ZC3HC1, RAN, PAPD7, SMC2, STRA13, MAD2L1, ZWINT, BRE, MAPRE2, HAUS7, RUVBL1, ARL8B, MAD2L2, SMC1A, NUP43, TXNL4A* | 22,25 | 3,7E-28 |
| GO:0008283~cell proliferation | 19 | 21,6 | *MORF4L1, PTGES3, GNAI2, NASP, BYSL, SRA1, UBE2L3, PRDX1, PARK7, RPL29, GPX1, GNB1, GOLPH3, PRKAR1A, RAC1, CKS2, WDR12, YME1L1, PES1* | 17,75 | 2,3E-14 |
| GO:0006974~cellular response to DNA damage stimulus | 14 | 15,9 | *MORF4L1, CCNK, LIG1, PTTG1, RBX1, RPS3, STRA13, RAD21, BRE, NSMCE2, RUVBL1, CUL4B, MAD2L2, SMC1A* | 6,85 | 1,6E-04 |
| GO:0006281~DNA repair | 12 | 13,6 | *MORF4L1, STRA13, RAD21, LIG1, BRE, NSMCE2, CUL4B, RUVBL1, PTTG1, SMC1A, RPS3, RBX1* | 7,75 | 5,5E-04 |
| GO:0006260~DNA replication | 9 | 10,2 | *RBBP4, LIG1, NASP, TBRG1, MCM2, MCM3, MCM4, MCM5, MCM6* | 15,03 | 2,1E-04 |
| GO:0007059~chromosome segregation | 7 | 8,0 | *RAD21, MIS18A, NUP37, ARL8B, PTTG1, NUP43, RPS3* | 16,16 | 6,7E-03 |
| GO:0006270~DNA replication initiation | 5 | 5,7 | *MCM2, MCM3, MCM4, MCM5, MCM6* | 42,81 | 7,6E-03 |
| **Term** | **Count** | **%** | **Genes highly expressed in large B compared to small B** | **Fold Enrichment** | **FDR** |
| GO:0055114~oxidation-reduction process | 50 | 30,9 | *UQCRC2, LDHA, PDHB, HIBADH, MTHFD1, NDUFS7, MTHFD2, NDUFS5, IDH3G, NDUFS4, NDUFS8, NDUFS2, IMPDH2, NDUFB11, CYCS, QDPR, GRHPR, NDUFA12, NDUFA11, ACADVL, SQLE, RRM1, ALDH9A1, NDUFB3, NDUFB5, NDUFB6, NDUFB8, NDUFB9, HSD17B12, HADHA, IDH1, TSTA3, NDUFA4, NDUFA5, NDUFA2, NDUFA3, NDUFA8, NDUFA9, NDUFA6, NDUFA1, IDH3A, NDUFV3, ADI1, SDHB, AKR1B3, NDUFV1, SDHC, SDHD, NDUFV2, ALDH2* | 8,31 | 2,6E-28 |
| GO:0006810~transport | 46 | 28,4 | *UQCRC2, NDUFB3, NDUFB5, NDUFB6, TUSC3, NDUFB8, NDUFB9, ATP5G2, ATP6V1B2, ATP6V1G1, ATP5G1, ATP5G3, NDUFS7, GOT2, ATP6V0C, NDUFS5, ATP6V0E, NDUFS4, NDUFS8, ATP5L, PAFAH1B1, NDUFS2, ATP5K, ATP5J, NDUFA4, NDUFA5, NDUFA2, NDUFB11, NDUFA3, NDUFA8, NDUFA9, NDUFA6, ATP6V1D, NDUFA1, NDUFA12, NDUFA11, ATP6V1C1, NDUFV3, SDHB, ATP6V1E1, SDHC, NDUFV1, SDHD, NDUFV2, ATP5C1, SCP2* | 2,84 | 1,5E-07 |
| GO:0008152~metabolic process | 33 | 20,4 | *CNDP2, HMGCS1, PGAM1, ECHS1, ACAT1, PDHB, HADHA, MTHFD1, MTHFD2, GALK1, PGP, ACSS1, IDS, NT5C3, UCK2, TSTA3, SUCLA2, AGPAT2, ACSL5, ACAA2, SUCLG1, EPRS, TKT, GRHPR, ACADVL, UMPS, RRM1, ALDH2, SMPD1, DNMT1, SCP2, ALDH9A1, PYGB* | 8,00 | 1,2E-16 |
| GO:0006629~lipid metabolic process | 15 | 9,3 | *PTGES3, ACAA2, CRLS1, PLA2G16, CDIPT, HSD17B12, HMGCS1, FDPS, ECHS1, HADHA, ACADVL, PAFAH1B1, PAFAH1B2, AGPAT2, ACSL5* | 3,67 | 8,8E-02 |
| GO:0015992~proton transport | 14 | 8,6 | *ATP5G2, ATP5G1, ATP6V1G1, ATP6V1B2, ATP6V1D, ATP5G3, ATP6V0C, ATP6V1C1, ATP6V0E, ATP6V1E1, ATP5C1, ATP5L, ATP5K, ATP5J* | 27,11 | 4,1E-12 |
| GO:0006099~tricarboxylic acid cycle | 12 | 7,4 | *SDHB, IDH3G, SUCLG1, SDHC, CS, SDHD, IDH3B, IDH1, FH1, SUCLA2, PDHB, IDH3A* | 46,47 | 8,2E-13 |
| GO:0015991~ATP hydrolysis coupled proton transport | 8 | 4,9 | *ATP6V1C1, ATP6V0C, ATP6V0E, ATP6V1E1, ATP5G2, ATP6V1B2, ATP5G1, ATP5G3* | 27,23 | 1,9E-05 |
| GO:0046034~ATP metabolic process | 8 | 4,9 | *ATP5C1, AK2, ATP5L, ATP6V1B2, ATP5G1, GUK1, ATP5K, ATP5J* | 22,46 | 8,0E-05 |
| GO:0009116~nucleoside metabolic process | 7 | 4,3 | *UMPS, UPRT, HPRT, PNP2, PNP, PRPS2, PPAT* | 39,31 | 2,3E-05 |
| GO:0015986~ATP synthesis coupled proton transport | 7 | 4,3 | *ATP5C1, ATP5L, ATP5G2, ATP5G1, ATP5G3, ATP5K, ATP5J* | 34,18 | 5,8E-05 |
| GO:0006123~mitochondrial electron transport, cytochrome c to oxygen | 6 | 3,7 | *COX8A, CYCS, COX4I1, COX6A1, COX5A, COX5B* | 56,16 | 5,7E-05 |
| GO:0006163~purine nucleotide metabolic process | 5 | 3,1 | *NME2, NME3, NME1, ADSL, GUK1* | 70,19 | 5,9E-04 |
| GO:0006183~GTP biosynthetic process | 5 | 3,1 | *NME2, NME3, NME1, PNP, IMPDH2* | 51,05 | 2,7E-03 |
| GO:0009142~nucleoside triphosphate biosynthetic process | 5 | 3,1 | *NME2, NME3, NME1, SUCLG1, CMPK1* | 46,80 | 4,1E-03 |
| GO:0006103~2-oxoglutarate metabolic process | 5 | 3,1 | *GOT2, IDH3G, IDH3B, IDH1, IDH3A* | 31,20 | 2,4E-02 |
| GO:1902600~hydrogen ion transmembrane transport | 5 | 3,1 | *ATP6V0E, COX8A, ATP5G2, ATP5G1, ATP5G3* | 29,56 | 3,1E-02 |
| GO:0006164~purine nucleotide biosynthetic process | 5 | 3,1 | *MTHFD1, HPRT, ADSL, GMPS, IMPDH2* | 28,08 | 3,8E-02 |
| **Term** | **Count** | **%** | **Genes highly expressed in small B compared to large B** | **Fold Enrichment** | **FDR** |
| GO:0042742~defense response to bacterium | 14 | 15,2 | *DEFA25, DEFA24, DEFA23, LYZ2, DEFA22, LYZ1, DEFA21, MMP7, NAIP6, DEFA5, DEFA26, DEFA3, DEFA2, DEFA17* | 19,49 | 2,9E-10 |
| GO:0006952~defense response | 7 | 7,6 | *DEFA25, DEFA24, DEFA22, AY761184, DEFA21, DEFA5, DEFA26* | 16,05 | 6,2E-03 |
| GO:0050829~defense response to Gram-negative bacterium | 6 | 6,5 | *LYZ2, LYZ1, MMP7, DEFA3, DEFA2, DEFA17* | 28,49 | 2,8E-03 |
| GO:0050830~defense response to Gram-positive bacterium | 6 | 6,5 | *LYZ2, LYZ1, MMP7, DEFA3, DEFA2, DEFA17* | 17,16 | 3,4E-02 |

Table S2 Gene-ontology-enrichment-score (functional cluster analysis) of highly repressed genes by *L. reuteri*-treatment in large-B.

| **Annotation Cluster 1 Enrichment Score: 4.53** | | | | |  |  |
| --- | --- | --- | --- | --- | --- | --- |
| **Category** | **Term** | **Count** | **%** | **Genes** | **Fold Enrichment** | **FDR** |
| UP_KEYWORDS | Cell cycle | 20 | 10,4 | *PPP6C, CCNK, 2610002M06RIK, LIG1, TPX2, PAPD5, CDC20, MCM2, CLTC, MCM5, SMC3, PPM1G, CDCA8, CASP8AP2, PLK1, USP39, RUVBL1, SMC1A, CCNA2, SEPT9* | 3,85 | 1,4E-03 |
| GOTERM_BP_DIRECT | GO:0007049~cell cycle | 19 | 9,9 | *PPP6C, CCNK, 2610002M06RIK, LIG1, TPX2, PAPD5, CDC20, MCM2, MCM5, SMC3, PPM1G, CDCA8, CASP8AP2, PLK1, USP39, RUVBL1, SMC1A, CCNA2, SEPT9* | 3,25 | 3,5E-02 |
| UP_KEYWORDS | Cell division | 16 | 8,3 | *CCNK, 2610002M06RIK, LIG1, TPX2, PAPD5, CDC20, CLTC, SMC3, MCM5, CDCA8, PLK1, USP39, RUVBL1, SMC1A, CCNA2, SEPT9* | 5,19 | 6,4E-04 |
| GOTERM_BP_DIRECT | GO:0051301~cell division | 15 | 7,8 | *CCNK, 2610002M06RIK, LIG1, TPX2, PAPD5, CDC20, SMC3, MCM5, CDCA8, PLK1, USP39, RUVBL1, SMC1A, CCNA2, SEPT9* | 4,22 | 2,1E-02 |
| UP_KEYWORDS | Mitosis | 11 | 5,7 | *CCNK, CDCA8, PLK1, TPX2, PAPD5, CDC20, RUVBL1, SMC1A, CLTC, CCNA2, SMC3* | 5,12 | 7,7E-02 |
| GOTERM_BP_DIRECT | GO:0007067~mitotic nuclear division | 11 | 5,7 | *CCNK, CDCA8, PLK1, TPX2, PAPD5, CDC20, RUVBL1, SMC1A, CLTC, CCNA2, SMC3* | 4,17 | 5,0E-01 |
| KEGG_PATHWAY | mmu04110:Cell cycle | 7 | 3,6 | *PLK1, CDC20, MCM2, SMC1A, CCNA2, SMC3, MCM5* | 4,23 | 7,0E+00 |

**Table S3** Primers used for quantitative-RT-PCR and oligonucleotides.

| **Gene Name** | **Forward primer (5’-3’)** | **Reverse primer (5’-3’)** |
| --- | --- | --- |
| ***TLR2*** | GGGGTGTGTGATGGCCGCTC | TGGAGGTTCGCACACGCTCG |
| ***TLR4*** | TGCTACAGCTCACCTGGGGCT | TCTGCCCGGTAAGGTCCATGC |
| ***TLR7*** | TGATCCTGGCCTATCTCTGAC | CGTGTCCACATCGAAAACAC |
| ***TLR9*** | GAGAATCCTCCATCTCCCAAC | CCAGAGTCTCAGCCAGCAC |
| ***CD1D*** | GCAGCCAGTACGCTCTTTTC | ACAGCTTGTTTCTGGCAGGT |
| ***S1PR1*** | GGAGGTTAAAGCTCTCCGC | CGCCCCGATGTTCAAC |
| ***S1PR2*** | GCAGTGACAAAAGCTGCCGAATGCTGATG | AGATGGTGACCACGCAGAGCACGTAGTG |
| ***CD69*** | TGGTCCTCATCACGTCCTTAATAA | TCCAACTTCTCGTACAAGCCTG |
| ***CXCR5*** | ACTCCTTACCACAGTGCACCTT | GGAAACGGGAGGTGAACCA |
| ***CCR6*** | CTGGAGCCGAGTGAGGTC | CGGCAAGCATTGTTCTCC |
| ***CCR9*** | CAATCTGGGATGAGCCTAAACAAC | ACCAAAAACCAACTGCTGCG |
| ***EBI2/GPR183*** | GACATCCTGTTTACCACAGCT | AGACCAGAATCCAGACGGACA |
| ***ITGA4*** | GTTATCCCTCTCCTCCAGGC | ACAGATGCGGGATCAGAAAG |
| ***ITGB7*** | TGTGCATGGTGCAAACAAC | GCGAGCCAGTAGCTCCTCT |
| ***AICDA*** | CGTGGTGAAGAGGAGAGATAGTG | CAGTCTGAGATGTAGCGTAGGAA |
| ***BAFFR*** | GAAACTGCGTGTCCTGTGAG | CTGAGGCTGCAGAGCTGTC |
| ***BCL-2*** | CTGGTGGACAACATCGCTCTG | GGTCTGCTGACCTCACTTGTG |
| ***BCMA*** | ACTTGCGATGTTCCAACCCT | ACTTGCGATGTTCCAACCCT |
| ***BLIMP-1*** | GAACCTGCTTTTCAAGTATGCTG | AGTGTAGACTTCACCGATGAGG |
| ***CD274*** | TAATCAGCTACGGTGGTGCG | CTTCTCTTCCCACTCACGGG |
| ***CD40*** | CCATGTGACTCAGGCGAAT | TAACCCGAAGCCCTTGATT |
| ***ICOSL*** | CGTCCCCACCGAAGCTATAC | GGGGGTCCACTCTGAAGTTG |
| ***TACI*** | GAGCTCGGGAGACCACAG | TGGTCGCTACTTAGCCTCAAT |
| ***TGFβ1*** | TGGAGCAACATGTGGAACTC | GTCAGCAGCCGGTTACCA |
| ***Sgpp1*** | CCCATTGGTGGACCTGATTG | GATGAGCGGCGCATATTTG |
| ***Sgpp2*** | CTATTACCTGTTCCGGTTTTCAGC | CTCTTTTCAAGTCTCACAACGGG |
| ***Spns2*** | GCACTTTGGGGTCAAGGA | CCCAGGTAGCCAAAGATGG |
| ***Sphk1*** | TCTGGGCTGCGGCTCTATT | AGGTCCACGTCAGCAACAAAG |
| ***Sphk2*** | CGGCCCACGGTTTGC | GGGCGTAGTCGCTGTATGTGT |
| ***Sgpl1*** | GTTGGGCCGCCTTGATG | GATGATCTGTTTGGTAGCTTCAACA |
| ***LASS5*** | GCAATGGTGCCAACTGCAT | TCCCCTGCTCTTCAGCCA |
| ***αGT*** | CCAGGCTAGACAGAGGCAAG | CGGAAGGGAAGTAATCGTGA |
| ***MyD88*** | CACCTGTGTCTGGTCCATTG | CTGTTGGACACCTGGAGACA |
| ***GAPDH*** | TGTGTCCGTCGTGGATCTGA | CCTGCTTCACCACCTTCTTGAT |
| ***TBP*** | CCCACCAGCAGTTCAGTAGC | CAATTCTGGGTTTGATCATTCTG |
| ***36B4*** | ACTGGTCTAGGACCCGAGAAG | CTCCCACCTTGTCTCCAGTC |
| **Oligonucleotides** | | |
| **oVPL3303** | ACCGTTATTCGATGGTGCAG | |
| **oVPL3304** | CAATCAGTAGTCGTTTGCGGAGTAC | |
| **oVPL3305** | AACTGGTAACGATGAAGATCTTGC | |
| **oVPL3306** | CAATCGCCCAATTTCATCAT | |
| **oVPL3307** | AAACGACGGCCAGTGAATTCGAGCTCGGTAACCGTTATTCGATGGTGCAGTTGAACTGCT | |
| **oVPL3308** | AGCAAGTACTCCGCAAACGACTACTGATTGAACTGGTAACGATGAAGATCTTGCATTACT | |
| **oVPL3309** | AAGTATATTAATGATGAAATTGGGCGATTGATCCTCTAGAGTCGACCTGCAGGCATGCAA | |
| **oVPL3310** | CGCTTCTGCTTCTGAGTTGA | |
| **oVPL3311** | GCCATGGTCTTGGCAATAAT | |
| **oVPL49** | ACAATTTCACACAGGAAACAGC | |
| **oVPL97** | CCCCCATTAAGTGCCGAGTGC | |
| **oVPL187** | TACCGAGCTCGAATTCACTGG | |
| **oVPL188** | ATCCTCTAGAGTCGACCTGC | |

Table S4 Histological scoring system for DSS induced ileal disruption.

| **Mucosal architecture** | **Score 1** | **Epithelial changes** | **Score 2** | **Inflammatory cell infiltrate** | **Score 3** |
| --- | --- | --- | --- | --- | --- |
|  | 0 |  | 0 |  | 0 |
| Mild villus blunting | 1 |  |  | Mild (mucosa) | 1 |
| Moderate villus blunting | 2 | Few erosions | 2 | Mild (mucosa + submucosa) | 2 |
| Moderate villus blunting +few villus atrophy | 3 | Erosions + ulcerations | 3 | Moderate (mucosa + submucosa + few transmural) | 3 |
| Villus atrophy+ distortion | 4 | Ulceration + necrosis/fibrosis | 4 | Marked (mucosa + submucosa + transmural) | 4 |

For the assessment of the severity of intestinal disruption, histological scores of the distal ileum were determined, and the sum was calculated (0-12)
